# Effect of High-Dose vs Standard-Dose Vitamin D Supplementation on Neurodevelopment of Healthy Term Infants: A Randomized Clinical Trial

**DOI:** 10.1001/jamanetworkopen.2021.24493

**Published:** 2021-09-08

**Authors:** Soile Tuovinen, Katri Räikkönen, Elisa Holmlund-Suila, Helena Hauta-alus, Otto Helve, Jenni Rosendahl, Maria Enlund-Cerullo, Eero Kajantie, Saara Valkama, Heli Viljakainen, Outi Mäkitie, Sture Andersson, Kati Heinonen

**Affiliations:** 1 Psychology/Welfare Sciences, Faculty of Social Sciences, University of Tampere, Tampere, Finland; 2 Department of Psychology and Logopedics, Faculty of Medicine, University of Helsinki, Helsinki, Finland; 3 Children’s Hospital, Pediatric Research Center, University of Helsinki, Helsinki University Hospital, Helsinki, Finland; 4 Research Program for Clinical and Molecular Metabolism, Faculty of Medicine, University of Helsinki, Helsinki, Finland; 5 Finnish Institute for Health and Welfare, Helsinki, Finland; 6 Research Unit for Pediatrics, Pediatric Neurology, Pediatric Surgery, Child Psychiatry, Dermatology, Clinical Genetics, Obstetrics and Gynecology, Otorhinolaryngology, and Ophthalmology, Medical Research Center Oulu, Oulu University Hospital, University of Oulu, Oulu, Finland; 7 Folkhälsan Research Center, Helsinki, Finland; 8 Department of Clinical and Molecular Medicine, Norwegian University for Science and Technology, Trondheim, Norway; 9 Center for Molecular Medicine, Karolinska Institutet and Clinical Genetics, Karolinska University Hospital, Stockholm, Sweden

## Abstract

**Importance:**

Vitamin D may be important for neurodevelopment. The optimal daily dose of vitamin D for early brain development is not known.

**Objectives:**

To test whether a higher (1200 IU) vs standard (400 IU) dose of vitamin D_3_ has beneficial effects on neurodevelopment in the first 2 years of life and whether serum 25-hydroxyvitamin D concentration is associated with neurodevelopment.

**Design, Setting, and Participants:**

This double-blind, interventional randomized clinical trial involved healthy infants born full-term between January 1, 2013, and June 30, 2014, at a maternity hospital in Helsinki, Finland, at the 60th northern latitude. Two-year follow-up was conducted by May 30, 2016. Data analysis was by the intention-to-treat principle. Data were analyzed from November 1, 2020, to May 31, 2021.

**Interventions:**

Randomization of 404 infants to receive 400 IU of oral vitamin D_3_ supplementation daily and 397 infants to receive 1200 IU of oral vitamin D_3_ supplementation daily from 2 weeks to 24 months of age.

**Main Outcomes and Measures:**

Primary outcomes were child total developmental milestone scores at 12 and 24 months of age measured using the Ages and Stages Questionnaire (total score is calculated as a mean of the 5 subscale scores: total score range, 0-60, where 0 indicates delay in all developmental domains and 60 indicates that the child can master all age-specific skills) as well as externalizing, internalizing, and dysregulation problems and competencies scores at 24 months measured using the Infant-Toddler Social and Emotional Assessment (range 0-2, where 0 indicates no problems or no competencies and 2 indicates a high level of problems or a high level of competencies; variables were standardized to the mean [SD] of 0 [1]). Secondary outcomes were specific skills, problems, and competencies derived from these questionnaires.

**Results:**

Of the 987 families recruited, 495 children were randomly assigned to receive 400 IU of vitamin D_3_, and 492 children were randomly assigned to receive 1200 IU of vitamin D_3_. A total of 801 families participated in the follow-up at 12 and/or 24 months, with 404 children (207 girls [51.2%]) in the 400-IU group and 397 children (198 girls [49.9%]) in the 1200-IU group. All children were of Northern European ethnicity. No differences were found between the 400-IU group and the 1200-IU group in the mean (SD) adjusted Ages and Stages Questionnaire total score at 12 months (45.0 [7.1] vs 46.2 [7.9]; mean difference [MD], 1.17 [95% CI, –0.06 to 2.38]) or 24 months (50.9 [5.3] vs 51.5 [5.5]; MD, 0.48 [95% CI, –0.40 to 1.36]). No differences were found between the 400-IU group and the 1200-IU group at 24 months in the mean (SD) adjusted Infant-Toddler Social and Emotional Assessment externalizing domain score (–0.07 [1.00] vs 0.07 [0.98]; MD, 0.15 [95% CI, –0.01 to 0.31]), internalizing domain score (0.04 [1.06] vs –0.02 [0.98]; MD, –0.07 [95% CI, –0.24 to 0.1.0]), dysregulation domain score (–0.00 [1.04] vs 0.02 [0.96]; MD, 0.02 [95% CI, –0.14 to 0.18]), or competencies score (–0.02 [1.02] vs 0.01 [1.02]; MD, 0.03 [95% CI, –0.13 to 0.20]). The 1200-IU group did have a higher risk in the adjusted model of scoring 1.5 SDs or more on the externalizing domain score (odds ratio, 2.33 [95% CI, 1.19-4.56]; *P* = .01). Levels of serum 25-hydroxyvitamin D were not associated with the primary outcomes.

**Conclusions and Relevance:**

Higher-than-standard vitamin D_3_ doses provide no systematic benefits for child neurodevelopment up to 2 years of age. However, the potential disadvantageous effects of higher doses could not be fully excluded; even if minimal, the potential nonbeneficial effects of higher-than-standard doses warrant further studies in which both safety and benefits should be evaluated.

**Trial Registration:**

ClinicalTrials.gov Identifier: NCT01723852

## Key Points

**Question:**

Does a higher (1200 IU) daily dose of vitamin D_3_ administered to healthy term infants between 2 weeks and 24 months of age have beneficial effects on neurodevelopment compared with the standard dose (400 IU) in Finland at the 60th northern latitude?

**Findings:**

This randomized clinical trial conducted among 801 families found no systematic difference in developmental milestones or social-emotional problems and competencies acquisition between the intervention groups up to 24 months of age.

**Meaning:**

These findings suggest that higher-than-standard doses of vitamin D are not required to optimize child neurodevelopment.

## Teaser

This randomized clinical trial tests whether a higher (1200 IU) vs standard (400 IU) dose of vitamin D_3_ has beneficial effects on neurodevelopment in the first 2 years of life and whether serum 25-hydroxyvitamin D concentration is associated with neurodevelopment.

## Introduction

Vitamin D is a steroid hormone with an acknowledged role in bone health.^1,2^ In addition, it has diverse extraskeletal roles in neurologic, immune, and inflammatory disorders.^3,4^ Vitamin D also has an important role in the development and function of the nervous system; vitamin D receptors have been identified in different parts of the brain,^5^ and animal and in vitro studies indicate that vitamin D affects structural brain development, neuroprotection, and neurotrophic functions.^3,6^ Hence, vitamin D may be important for neurodevelopment, especially in the early years of life when the brain is developing rapidly and is sensitive to nutrient deficiencies.^7^

Vitamin D status is best defined as the blood serum 25-hydroxyvitamin D (25[OH]D) concentration. The current recommendations of optimal serum 25(OH)D concentration levels are based on studies on rickets and bone mass development,^1,8^ and, to our knowledge, the optimal levels for brain development are not known. Although there is a global consensus to give a moderate (10 μg [400 IU]) daily dose of vitamin D supplementation to all children^8,9,10^ and many countries have also implemented guidelines for vitamin D food fortification,^11^ vitamin D inadequacy (25[OH]D level <20.03 ng/mL [to convert to nanomoles per liter, multiply by 2.496])^12^ has been a worldwide health concern, particularly among children.^13^

Systematic reviews and meta-analyses summarize previous findings and suggest that lower vitamin D concentrations in childhood are associated with neurodevelopmental disorders, including autism spectrum disorder and attention-deficit/hyperactivity disorder in children.^14,15^ Associations between vitamin D concentrations and cognitive and motor functioning have not been systematically found.^16^ However, previous evidence is based on observational studies, precluding causal inferences. Randomized clinical trials (RCTs),^17,18,19,20^ as well as nonrandomized trials,^21,22,23,24^ on vitamin D supplementation for children have been small, have typically focused on symptom severity among children with autism spectrum disorder or attention-deficit/hyperactivity disorder, and have reported mixed findings. We are aware of only 1 RCT on vitamin D supplementation that has focused on child neurodevelopment.^25^ This trial randomized 55 children to receive 400 IU, 800 IU, and 1200 IU of vitamin D daily from the age of 2 weeks onward and showed that, at the age of 3 months, the groups did not differ on motor development, but at the age of 6 months, the children who received the lowest daily dose had higher motor development scores than those who received higher daily doses.^25^

In a large, community-based sample of healthy children born full-term, we tested whether those randomly assigned to receive 400 IU and 1200 IU of vitamin D_3_ supplementation daily between 2 weeks and 24 months of age differed in their acquisition of age-appropriate developmental milestones at 12 and 24 months of age and in social-emotional problems and competencies at 24 months of age. We also tested whether the children’s serum levels of 25(OH)D at 12 and 24 months of age were associated with these child neurodevelopmental outcomes. We hypothesized that higher dosages of vitamin D_3_ supplementation and higher 25(OH)D levels would lead to higher developmental milestone scores, a lower risk of mild developmental delay, less social-emotional problems, more competencies, a lower risk of clinically relevant problems, and a lower risk of a delay in competencies.

## Methods

### Study Design and Participants

The Vitamin D Intervention in Infants (VIDI) study is a double-blind, interventional randomized clinical trial.^26,27^ Infants began receiving vitamin D_3_ supplementation at 2 weeks of age and continued until 24 months of age. Between January 1, 2013, and June 30, 2014, 987 families were recruited at Kätilöopisto Maternity Hospital, Helsinki, Finland, at the 60th northern latitude. Two-year follow-up was conducted by May 30, 2016. The trial protocol (Supplement 1) has been described previously.^26,27,28^ The participating children’s parents signed an informed consent form at recruitment. Previous intervention studies have evaluated the safety of substitution doses of up to 50 μg (2000 IU) of vitamin D in infants,^29^ and the safety of substitution doses of 400, 1200, and 1600 IU was also ensured in a pilot study for VIDI.^30^ No hypercalcemia was observed, and in all intervention groups, the mean 25(OH)D concentration reached at least 32 ng/mL. The maximum 25(OH)D concentrations in the 400-IU, 1200-IU, and 1600-IU intervention groups were 50, 79.2, and 92 ng/mL, respectively, after 3 months of supplementation. For safety reasons, to avoid unnecessarily high levels of 25(OH)D, the 1600-IU group was not included in the full-scale RCT. An external steering group was recruited to monitor the study. The vitamin D_3_ supplements were prepared by Orion Pharmaceuticals. The study is researcher initiated and independent. The research ethics committee of the Hospital District of Helsinki and Uusimaa has approved the study, and it is registered with ClinicalTrials.gov (NCT01723852). The study is reported according to the Consolidated Standards of Reporting Trials (CONSORT) reporting guideline.

### Procedure

Infants (492 girls and 495 boys) were randomized on a 1:1 basis to receive 400 IU (10 μg) or 1200 IU (30 μg) of vitamin D_3_ daily from 2 weeks to 24 months of age. The 400-IU and 1200-IU groups did not differ in mean (SD) maternal pregnancy 25(OH)D concentrations (82.5 [22.0] vs 82.1 [17.9] ng/mL; *t* = –0.30; *P* = .76). Vitamin D was administered once daily with 5 drops for both groups.

Data on developmental milestones were collected at 12 and 24 months of age, and data on social-emotional problems and competencies were collected at 24 months of age. Of the 987 recruited families, 12 did not meet the inclusion criteria. A total of 801 families participated in the follow-up at 12 and/or 24 months and were included in the current study (Figure).^26^ Of those families, 667 completed a questionnaire on child developmental milestones at the child’s mean (SD) age of 11.6 (0.4) months (range, 10.5-13.1 months), and 636 families completed a questionnaire on child developmental milestones at the child’s mean (SD) age of 23.5 (0.4) months (range, 22.3-25.1 months). Furthermore, 657 families completed a questionnaire on child social-emotional problems and competencies at the child’s mean (SD) age of 25.8 (2.4) months (range, 12.6-36.9 months). Comparisons of each analytic sample against cohort members who could not be included owing to missing data (attrition group) are shown in eTable 1 in Supplement 2.

**Figure.**
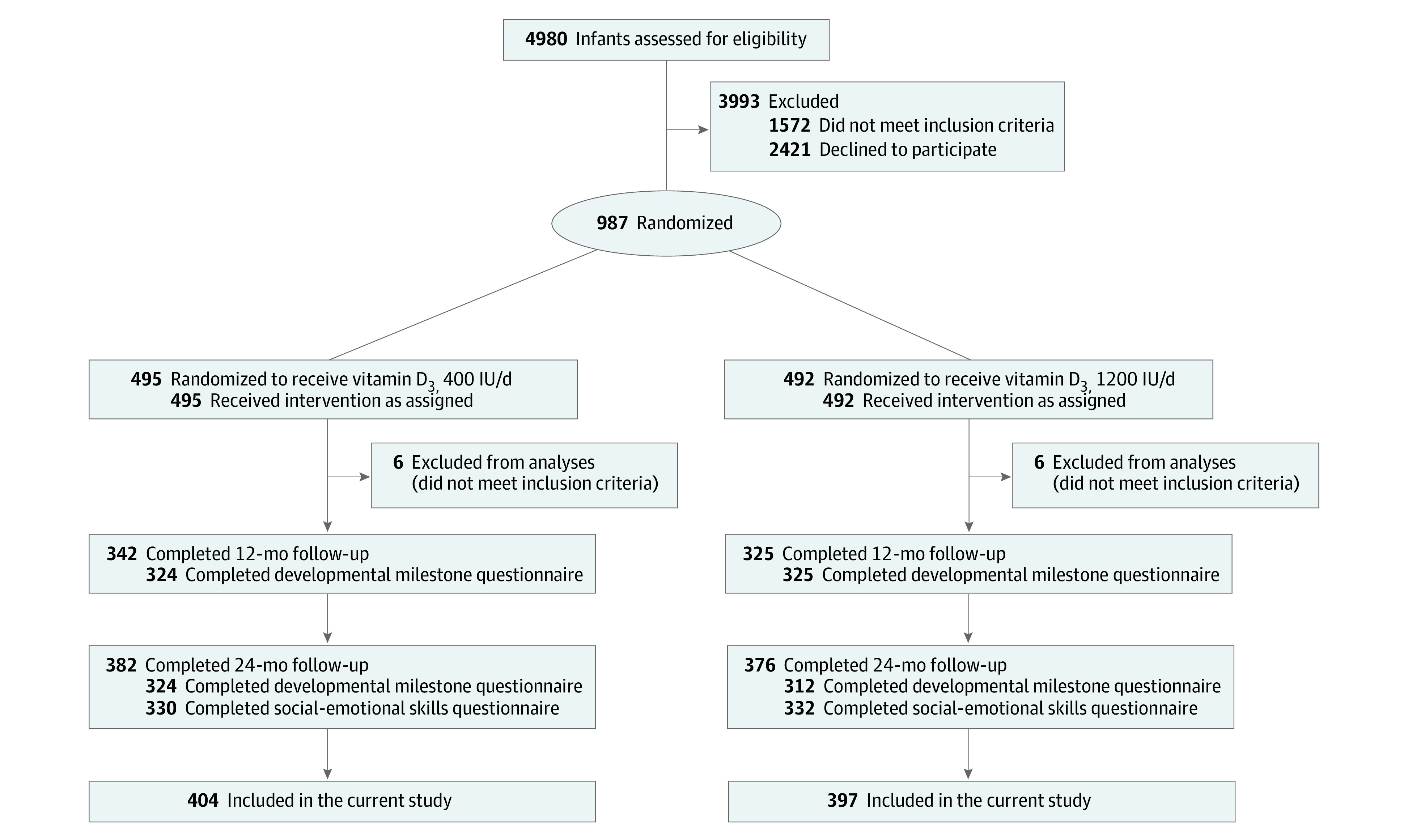
Flowchart of the Study Enrollment, Allocation, and Follow-up

### Biochemical Analyses

Serum samples of 25(OH)D were obtained from each child at the 12- and 24-month follow-up visits with a fully automated immunoassay (IDS-iSYS; Immunodiagnostic System Ltd). Details on laboratory analyses are provided in eAppendixes 1 and 2 in Supplement 2 and are described elsewhere.^26,27^

### Outcome Measures

#### Developmental Milestones

We assessed developmental milestones with the Ages and Stages Questionnaire (ASQ), third edition (total score is calculated as a mean of the 5 subscale scores: total score range, 0-60, where 0 indicates delay in all developmental domains and 60 indicates that the child can master all age-specific skills). The ASQ is a reliable and valid tool with high sensitivity and specificity for screening children requiring further developmental assessment.^31,32^ The ASQ measures communication, problem solving, gross motor skills, fine motor skills, and personal and social skills and comprises 30 age-appropriate items scored 10 if the child can master the skill, 5 if the skill is emerging or occasional, and 0 if the child cannot master the skill.^32^ The ASQ total score, calculated as a mean of the subscale scores, was used as the primary outcome. As secondary outcomes, we used the 5 raw domain-specific scores. We allowed 30% deviation from the 11- to 13-month and 23- to 25.5-month questionnaire-specific age.^33,34^

#### Social-Emotional Problems and Competencies

We assessed social-emotional problems and competencies with the Infant-Toddler Social and Emotional Assessment (ITSEA) (range 0-2, where 0 indicates no problems or no competencies and 2 indicates a high level of problems or a high level of competencies; variables were standardized to the mean [SD] of 0 [1]).^35^ It includes 169 items containing a statement about the child’s behavior during the last month. The scale has good psychometric properties.^35^

As primary outcomes, we used the scores from the 4 core ITSEA domains: externalizing (24 items, Cronbach α = 0.81; measuring activity, impulsivity, aggression, and defiance), internalizing (32 items, Cronbach α = 0.78; measuring depression, withdrawal, anxiety, separation distress, and inhibition), dysregulation (34 items, Cronbach α = 0.83; measuring problems in sleeping, eating, emotional reactivity and regulation, and unusual sensory sensitivities), and competence (37 items, Cronbach α = 0.83; measuring compliance, attention regulation, imitation and pretend play skills, mastery motivation, empathy, emotional awareness, and prosocial peer behaviors).^35^ As secondary outcomes, we used the specific problems and competencies subscales (described under domains). More detailed descriptions of the outcome measures are provided in eAppendix 1 in Supplement 2.

### Covariates

Potential covariates known to be associated with vitamin D levels and/or neurodevelopment used in the current study were child’s sex,^36^ gestational length (weeks),^37^ season of birth,^38,39^ fetal cord blood 25(OH)D level,^40,41^ duration of breastfeeding, maternal age at delivery (in years),^42^ maternal smoking,^43^ depressive symptoms^44^ (measured using the Center for Epidemiological Studies Depression Scale^45^), and maternal educational level.^46^ Details of data collection and laboratory test analyses are provided in eAppendix 1 in Supplement 2.

### Statistical Analysis

Data analysis was by the intention-to-treat principle. Data were analyzed from November 1, 2020, to May 31, 2021. We compared baseline and follow-up characteristics with the 2-tailed independent sample *t* test or Pearson χ^2^ test.

We assessed differences between the intervention groups in the primary outcomes using Tobit regression (ASQ total scores), linear regression (ITSEA domain scores), and logistic regression (ASQ total and ITSEA domain scores). Tobit regression accounted for the ceiling effect resulting from the ASQ not distinguishing between children mastering all age-specific skills (in the current study, 7.2% [48 of 666] to 53.5% [340 of 636] of children mastered all age-specific skills). For logistic regression, the ASQ total score was dichotomized at −1 SD; the ITSEA internalizing, externalizing, and dysregulation scores were dichotomized at 1.5 SDs; and the competencies scale was dichotomized at −1.5 SDs based on rank-normalized values according to the Blom formula,^47^ indicating mild developmental delay^32,48^ and clinically relevant problems and delay in competencies.

Differences between the intervention groups in the secondary outcomes were tested using ordinal logistic regressions (ie, ASQ domain-specific scores because each ASQ item is measured ordinally, yielding subscale scores ranging from 0 to 60) and linear regressions (ITSEA subscale scores). Finally, we tested associations between 25(OH)D levels measured at 12 and 24 months of age and primary outcomes measured at corresponding follow-ups using the regressions already described.

For the 400-IU group and 1200-IU group comparisons for our primary outcomes with α = .05, we had 0.8 power to confirm or exclude effect sizes (Cohen *d*) greater than 0.11 in continuous variables and odds ratios (ORs) greater than 1.9 in dichotomous variables, assuming a 10% prevalence rate. As the effect size, we present mean differences (MDs), unstandardized regression coefficients (β), and ORs with their 95% CIs from a crude model and an adjusted model. The analyses regarding the secondary outcomes should be interpreted as exploratory owing to the potential of type I error associated with multiple comparisons. We made adjustments for covariates that were statistically significantly associated with 1 or more of the primary outcomes (eTable 2 in Supplement 2). There were no multicollinearity symptoms between the covariates (all variance inflation factors >1 and <1.3).

We also tested whether a child’s sex modified any of the potential associations between intervention groups or the levels of 25(OH)D and our primary outcomes by entering interactions terms “sex × intervention group, level of 25(OH)D” into the equations. We also conducted sensitivity analyses and repeated the primary analyses that tested the associations between intervention groups or 25(OH)D levels and developmental milestones, restricting the sample to those who responded to the questionnaire within the questionnaire-specific age range (646 at 12 months of age and 578 at 24 months of age).

We first set the significance level of α to .05. We then controlled for inflation of the type I error rate from multiple testing with a false detection rate procedure.^49^ We corrected for a 5% false detection rate over 6 tests for our primary outcomes and 27 tests for our secondary outcomes.

## Results

### Characteristics

Of the 801 families included in the current study, 405 children (50.6%) were girls, 596 children were born to 778 mothers (76.6%) with a high educational level, and all were of Northern European ethnicity. Table 1 presents the baseline characteristics and Table 2 presents the follow-up characteristics of the 801 children according to the intervention groups. The 400-IU and 1200-IU groups did not differ significantly in child or parental background characteristics. As expected, the mean (SD) 25(OH)D levels were significantly higher at both the 12-month and 24-month follow-ups in the 1200-IU group compared with the 400-IU group (12-month follow-up: 46.2 [11.0] vs 33.3 [7.9] ng/mL; 24-month follow-up: 47.3 [10.6] vs 34.8 [7.8] ng/mL) (Table 2). Of all participants, 98.9% (738 of 746) were vitamin D sufficient (25[OH]D level >20.0 ng/mL) at the 12-month follow-up and 99.5% (764 of 768) were vitamin D sufficient at the 24-month follow-up. No participants had vitamin D toxicity (indicated by a 25(OH)D level ≥100 ng/mL). eTable 2 in Supplement 2 shows the associations of the covariates with the primary outcomes.

**Table 1.** Baseline Characteristics of the Participants by the Intervention Group^a^

Characteristic	400-IU group (n = 404), No. (%)	No.	1200-IU group (n = 397), No. (%)	No.
**Child**				
At birth				
Female sex	207 (51.2)	404	198 (49.9)	397
Length of gestation, mean (SD), d	280.7 (7.7)	404	281.5 (7.5)	397
Season of birth		404		397
Winter	83 (20.5)		72 (18.1)	
Spring	166 (41.1)		167 (42.1)	
Summer	85 (21.0)		90 (22.7)	
Autumn	70 (17.3)		68 (17.1)	
25(OH)D concentration at birth				
Cord blood, mean (SD) [range], ng/mL	33.3 (11.4) [14.7-113.5]	395	32.8 (9.5) [15.1-91.6]	388
≤20 ng/mL	9 (2.3)	395	19 (4.9)	388
**Mother**				
Age at delivery, mean (SD), y	31.0 (4.1)	398	31.5 (4.6)	397
Smoking at childbirth, yes	54 (13.7)	395	57 (14.6)	391
Depressive symptoms at childbirth, mean (SD), score^b^	12.1 (6.5)	350	12.0 (6.1)	352
Educational level, high^c^	292 (75.6)	386	304 (77.6)	392

Abbreviation: 25(OH)D, serum 25-hydroxyvitamin D.

SI conversion factor: To convert 25(OH)D to nanomoles per liter, multiply by 2.496.

^a^ Sample with data available on developmental milestones or social-emotional problems and competencies.

^b^ Depressive symptoms were measured with the Center for Epidemiological Studies Depression Scale.

^c^ Missing values were dummy-coded to their own category.

**Table 2.** Follow-up Characteristics of the Participants by the Intervention Group^a^

Characteristic	400-IU group (n = 404), mean (SD)	No.	1200-IU group (n = 397), mean (SD)	No.	χ^2^ or MD	*P* value
At 12-mo follow-up						
Breastfed, mo	10.5 (5.7)	396	10.9 (5.6)	392	MD = 1.05	.29
25(OH)D concentration						
Mean (SD) [range], ng/mL	33.3 (7.9) [14.8-56.0]	373	46.2 (11.0) [20.7-96.4]	373	MD = 12.9	<.001
≤20 ng/mL, No. (%)	8 (2.1)	373	0	373	χ^2^ = 8.09	.004
Age at completing ASQ, mo	11.6 (0.4)	342	11.6 (0.4)	325	MD = 0.03	.31
ASQ total score^b^^,^^c^	45.4 (7.3)	336	46.1 (7.7)	322	NA	NA
ASQ subscale score, median (IQR)						
Communication skills	45 (35-50)	337	45 (35-50)	322	NA	NA
Gross motor skills	50 (35-60)	337	50 (35-60)	322	NA	NA
Fine motor skills	50 (45-60)	341	50 (45-60)	325	NA	NA
Problem solving skills	55 (45-60)	341	55 (45-60)	325	NA	NA
Personal social skills	40 (35-50)	341	40 (35-50)	325	NA	NA
At 24-mo follow-up						
25(OH)D concentration						
Mean (SD) [range], ng/mL	34.8 (7.8) [17.0-61.4]	386	47.3 (10.6) [22.6-83.0]	381	MD = 12.5	<.001
≤20 ng/mL, No. (%)	4 (1.0)	386	0	381	χ^2^ = 3.97	.05
Age at completing ASQ, mo	23.5 (0.4)	324	23.5 (0.4)	312	MD = 0.01	.71
ASQ total score^b^^,^^c^	51.0 (5.2)	323	51.3 (5.5)	311	NA	NA
ASQ subscale score, median (IQR)						
Communication skills	55 (50-60)	323	55 (50-60)	312	NA	NA
Gross motor skills	60 (50-60)	324	60 (50-60)	312	NA	NA
Fine motor skills	55 (50-60)	324	55 (50-60)	312	NA	NA
Problem solving skills	50 (40-55)	324	50 (40-55)	311	NA	NA
Personal social skills	50 (45-55)	324	50 (45-55)	312	NA	NA
Age at completing ITSEA, mo^d^	25.9 (2.4)	321	25.7 (2.3)	322	MD = −0.15	.43
ITSEA domain scores^c^						
Externalizing domain	0.49 (0.22)	325	0.51 (0.23)	327	NA	NA
Internalizing domain	0.48 (0.22)	325	0.48 (0.20)	328	NA	NA
Dysregulation domain	0.44 (0.23)	326	0.45 (0.23)	330	NA	NA
Competencies	1.45 (0.22)	325	1.45 (0.22)	328	NA	NA

Abbreviations: 25(OH)D, serum 25-hydroxyvitamin D; ASQ, Ages and Stages Questionnaire; IQR, interquartile range; ITSEA, Infant-Toddler Social and Emotional Assessment; MD, mean difference; NA, not applicable.

SI conversion factor: To convert 25(OH)D to nanomoles per liter, multiply by 2.496.

^a^ Sample with data available on developmental milestones or social-emotional problems and competencies.

^b^ Total score calculated as a mean of ASQ domain scores.

^c^ Mean differences are shown in Tables 3 and 4.

^d^ Missing values are replaced with a mean of the sample in the analyses.

### Vitamin D Supplementation and Developmental Milestones

There were no differences between the 400-IU group and the 1200-IU group in the mean (SD) adjusted ASQ total score at 12 months (45.0 [7.1] vs 46.2 [7.9]; MD, 1.17 [95% CI, –0.06 to 2.38]) or 24 months (50.9 [5.3] vs 51.5 [5.5]; MD, 0.48 [95% CI, –0.40 to 1.36]) (Table 3). There were also no differences between the 2 groups in the odds of scoring below the −1-SD cutoff on this score at 12 or 24 months of age. At 12 months of age, ASQ subscale scores on communication and problems solving skills were higher in the 1200-IU group than in the 400-IU group across both unadjusted and adjusted models (eTable 3 in Supplement 2). The intervention groups did not differ in the other ASQ domain-specific scores (eTable 3 in Supplement 2).

**Table 3.** Associations Between Vitamin D Supplementation (1200 IU vs 400 IU) and Developmental Milestones^a^

Developmental milestone score	Mean (SD)		MD or OR (95% CI)	*P* value
	400-IU group (n = 322-336)	1200-IU group (n = 311-323)		
**At 12-mo follow-up**				
ASQ total score				
Model 1	45.3 (7.3)	46.1 (7.9)	MD, 0.77 (−0.39 to 1.93)	.20
Model 2	45.0 (7.1)	46.2 (7.9)	MD, 1.17 (−0.06 to 2.38)	.06
ASQ total score ≤ −1 SD, No. (%)				
Model 1	45 (13.4)	53 (16.5)	OR, 1.27 (0.83 to 1.96)	.27
Model 2	38 (13.6)	46 (16.7)	OR, 1.37 (0.85 to 2.20)	.20
**At 24-mo follow-up**				
ASQ total score				
Model 1	51.0 (5.2)	51.3 (5.5)	MD, 0.34 (−0.51 to 1.18)	.43
Model 2	50.9 (5.3)	51.5 (5.5)	MD, 0.48 (−0.40 to 1.36)	.29
ASQ total score ≤ −1 SD, No. (%)				
Model 1	44 (13.6)	49 (15.8)	OR, 1.19 (0.76 to 1.84)	.45
Model 2	39 (14.3)	40 (14.9)	OR, 1.18 (0.71 to 1.95)	.53

Abbreviations: ASQ, Ages and Stages Questionnaire; MD, mean difference; OR, odds ratio.

^a^ The MDs and 95% CIs from Tobit regression analyses refer to differences in ASQ total raw scores of 1200-IU group vs 400-IU group. OR and 95% CIs from logistic regression analyses show the odds of belonging to the group scoring –1 SD or less vs more than –1 SD in ASQ for 1200-IU group vs 400-IU group. All Cohen *d* effect sizes less than 0.11 in continuous variables. Model 1 is the crude model. Model 2 is adjusted for sex, length of gestation, duration of breastfeeding, age at follow-up, maternal age at delivery, maternal smoking and depressive symptoms at childbirth, and maternal educational level (missing values were dummy-coded to their own category). In model 2, 555 children were available at 12-month follow-up and 542 children were available at 24-month follow-up.

### Vitamin D Supplementation and Social-Emotional Problems and Competencies

No differences were found between the 400-IU group and the 1200-IU group at 24 months in the mean (SD) adjusted ITSEA externalizing domain score (–0.07 [1.00] vs 0.07 [0.98]; MD, 0.15 [95% CI, –0.01 to 0.31]), internalizing domain score (0.04 [1.06] vs –0.02 [0.98]; MD, –0.07 [95% CI, –0.24 to 0.1.0]), dysregulation domain score (–0.00 [1.04] vs 0.02 [0.96]; MD, 0.02 [95% CI, –0.14 to 0.18]), or competencies score (–0.02 [1.02] vs 0.01 [1.02]; MD, 0.03 [95% CI, –0.13 to 0.20]) (Table 4). However, the odds of scoring 1.5 SDs or more on the externalizing domain was statistically significantly higher for the 1200-IU group than for the 400-IU group in the adjusted model (OR, 2.33 [95% CI, 1.19-4.56; *P* = .01) (Table 4). The groups did not differ statistically significantly on the more specific problems or competencies scales (eTable 4 in Supplement 2).

**Table 4.** Associations Between Vitamin D Supplementation (1200 IU vs 400 IU) and Social-Emotional Problems and Competencies^a^

Social-emotional problems and competencies domain scores	Mean (SD)		MD or OR (95% CI)	*P* value
	400-IU group (n = 325-326)	1200-IU group (n = 327-330)		
Externalizing domain				
Model 1	−0.05 (0.99)	0.04 (1.01)	MD, 0.09 (−0.06 to 0.25)	.23
Model 2	−0.07 (1.00)	0.07 (0.98)	MD, 0.15 (−0.01 to 0.31)	.07
Externalizing domain score ≥1.5 SDs, No. (%)				
Model 1	19 (5.8)	32 (9.8)	OR, 1.75 (0.97 to 3.15)	.06
Model 2	14 (5.1)	31 (10.8)	OR, 2.33 (1.19 to 4.56)	.01
Internalizing domain				
Model 1	0.00 (1.04)	−0.01 (0.96)	MD, −0.02 (−0.17 to 0.14)	.85
Model 2	0.04 (1.06)	−0.02 (0.98)	MD, −0.07 (−0.24 to 0.10)	.43
Internalizing domain score ≥1.5 SDs, No. (%)				
Model 1	26 (8.0)	24 (7.3)	OR, 0.91 (0.51 to 1.62)	.74
Model 2	25 (9.1)	22 (8.1)	OR, 0.82 (0.45 to 1.50)	.52
Dysregulation domain				
Model 1	−0.02 (1.03)	0.01 (0.97)	MD, 0.04 (−0.11 to 0.19)	.62
Model 2	−0.00 (1.04)	0.02 (0.96)	MD, 0.02 (−0.14 to 0.18)	.82
Dysregulation domain score ≥1.5 SDs, No. (%)				
Model 1	13 (4.0)	18 (5.5)	OR, 1.39 (0.67 to 2.88)	.38
Model 2	13 (4.7)	16 (5.5)	OR, 1.25 (0.57 to 2.74)	.58
Competencies				
Model 1	0.00 (1.00)	−0.00 (1.01)	MD, −0.01 (−0.16 to 0.15)	.94
Model 2	−0.02 (1.02)	0.01 (1.02)	MD, 0.03 (−0.13 to 0.20)	.69
Competencies score ≤ –1.5 SDs, No. (%)				
Model 1	24 (7.4)	24 (7.3)	OR, 0.99 (0.55 to 1.78)	.97
Model 2	22 (8.0)	21 (7.3)	OR, 0.88 (0.47 to 1.65)	.69

Abbreviations: ITSEA, Infant-Toddler Social and Emotional Assessment; MD, mean difference; OR, odds ratio.

^a^ Values represent means in SD units and MDs with 95% CIs in ITSEA domain scores from linear regression analyses. OR and 95% CI from logistic regression analyses show the odds of belonging to the group scoring 1.5 or more SD vs less than 1.5 SD (problems domains) or –1.5 SD or less vs more than –1.5 SD (competencies domain) in ITSEA domains for 1200-IU group vs 400-IU group. To facilitate comparison of effect sizes all continuous outcome variables were standardized to the mean of 0 and SD of 1. Model 1 is the crude model. Model 2 is adjusted for sex, length of gestation, duration of breastfeeding, age at follow-up (missing values replaced with a mean of the sample), maternal age at delivery, maternal smoking and depressive symptoms at childbirth, and maternal educational level (missing values dummy-coded to their own category). In model 2, 274 children were in the 400-IU group and 286 to 287 children were in the 1200-IU group.

### Levels of 25(OH)D and Developmental Milestones and Social-Emotional Problems and Competencies

In both the unadjusted and adjusted models, 25(OH)D levels at 12 or 24 months of age were not statistically significantly associated with the primary outcomes (eTables 5 and 7 in Supplement 2) or secondary outcomes (eTables 6 and 8 in Supplement 2). Only in the adjusted model of the secondary outcomes were higher 25(OH)D levels associated with higher problem-solving skills (eTable 6 in Supplement 2). Furthermore, only in the unadjusted model were higher 25(OH)D levels associated with fewer sleeping and eating problems (eTable 8 in Supplement 2). These associations did not survive correction for multiple testing.

### Moderation by Sex and Sensitivity Analyses

Sex × intervention group or sex × 25(OH)D level interactions were not significant in any of the primary outcome analyses. Results of the sensitivity analyses among those whose developmental milestones were reported within the questionnaire specific-age range were mainly in line with the presented results. There were only 2 significant associations: at 12 months of age, the ASQ total score was higher in the 1200-IU group than in the 400-IU group in the adjusted model (β = 1.41 [95% CI, 0.17-2.66]; *P* = .03), and higher 25(OH)D levels were associated with higher ASQ total scores at 12 months of age in the adjusted model (β = 0.66 [95% CI, 0.02-1.31]; *P* = .05). Furthermore, consistent with earlier reported results, these associations did not survive correction for multiple testing.

## Discussion

We evaluated the effects of vitamin D supplementation with doses of 1200 IU and 400 IU on neurodevelopmental outcomes in an RCT among 801 healthy infants born full-term, most of whom had sufficient levels of vitamin D. We found no systematic differences in main neurodevelopmental outcomes—total developmental milestone scores and social-emotional problems and competencies domain scores—at 12 or 24 months of age between the vitamin D intervention groups. Our study had adequate power to detect, or exclude, small to medium differences in effect size between the groups.^50^

We observed that the children receiving 1200 IU of vitamin D supplementation had a higher risk of scoring 1.5 SDs or higher on the externalizing symptoms scale at 24 months after covariates were taken into account. Furthermore, of the secondary outcomes, we found that children receiving 1200 IU of vitamin D supplementation had better developmental milestone subscale scores in communication and problem-solving skills at 12 months. Furthermore, we found that higher 25(OH)D concentrations were associated with fewer sleeping problems at 24 months.

Most of the associations found had small effect sizes. We had multiple outcome variables, increasing the possibility that these associations arise by chance (type I error). All associations were rendered nonsignificant after correction for multiple testing (false detection rate) and/or adjustment for covariates. However, to our knowledge, there is limited research knowledge on the effects of high vitamin D levels and/or excessive supplementation on neurodevelopment. Our findings of the negative consequences warrant further studies on whether deficient or inadequate levels and also high levels of vitamin D are associated with adverse developmental outcomes. Furthermore, some effects of early intervention may only become apparent with age when higher-level cognitive and behavioral skills are required, thereby highlighting the importance for future studies.

The absence of children in our study who had deficient or inadequate vitamin D levels may explain the lack of any systematic effects of higher levels of vitamin D supplementation on our outcomes. This study was conducted in a population of northern latitude but where severe vitamin D inadequacy is rare owing to public health efforts, including food fortification and promotion of vitamin D supplementation.^9,51^ At birth, 755 of 783 children (96.4%) were vitamin D sufficient, and at 24 months of age, 764 of 768 children (99.5%) were vitamin D sufficient. Furthermore, for 635 of 763 participants (83.2%), 25(OH)D levels exceeded 30 ng/mL at 24 months of age, suggesting that both doses have been adequate in maintaining sufficient vitamin D status. Another possibility is that, among vitamin D–sufficient children, factors other than supplementation doses may have a greater influence on neurodevelopment. A further explanation is that the lack of positive effects from the higher-dose vitamin D supplementation on neurodevelopment is due to the timing of the intervention. Brain growth and development is pronounced during gestation and pregnancy.^52^ However, in line with our findings, a recent RCT of 2800 IU vs 400 IU of vitamin D_3_ supplementation during the third trimester of pregnancy showed no improvements in neurodevelopmental outcomes in the offspring during the first 6 years of life.^53^

Evidence has been controversial concerning associations between vitamin D supplementation and neurodevelopmental outcomes among children. Although not directly comparable, our findings are in line with 2 small-scale RCTs reporting a lack of association between vitamin D supplementation (compared with no supplementation or placebo) and autism spectrum disorder symptoms among children.^19,20^ Opposed to our findings, 4 previous studies have reported that vitamin D supplementation is associated with reduced symptoms among children with autism spectrum disorder^18,22,23^ or attention-deficit/hyperactivity disorder.^17,21^ However, none of these studies comprised a healthy population, and all of these RCTs were restricted to supplementation effects on either autism spectrum disorder or attention-deficit/hyperactivity disorder symptoms. To our knowledge, the only previous study (among 55 healthy Canadian infants with 25[OH]D levels comparable to ours) concluded that those receiving lower levels of vitamin D supplementation (400 IU vs 800 or 1200 IU) had higher gross motor achievements at 6 months of age.^25^ This finding is contrary to our lack of association between vitamin D dose and motor skills. In the Canadian study, children were examined at a younger age than in our study, which offers one possible explanation for the difference in findings.

### Strengths and Limitations

This study has some strengths, including a sizable homogenous sample; recruitment that took place in a single hospital, enabling standardized data collection; a double-blind, randomized clinical trial design; and a well-characterized sample. However, the study also has some limitations, including the homogeneity of the sample. The mothers in the sample were well educated, and all were of Northern European ethnicity, which may affect the generalizability of the findings. Also, the limited number of children with low 25(OH)D levels may have restrained our analyses. Furthermore, we had a single informant on child neurodevelopment. However, the methods used in our study are well validated.^31,35^

## Conclusions

In a country where sunlight exposure is limited but food fortification with vitamin D is common, 1200 IU vs 400 IU of vitamin D supplementation did not provide a benefit for healthy term infants’ developmental milestones or social-emotional skill acquisition. However, minimal signs of a potential negative impact of higher doses warrants further studies in which both the safety and the benefits should be considered.

## Notes

Supplement 1.
Trial Protocol

Supplement 2. **eAppendix 1.** Supplementary Methods
eReferences
**eAppendix 2.** The Report From Immunodiagnostic Systems Containing the Linear Regression Equation for Correction of Cord Blood 25-Hydroxyvitamin D Concentration **eTable 1.** Comparison of Analytic Sample Against Cohort Members Who Could Not Be Included Due to Missing Data (Attrition Group) **eTable 2.** Associations Between Covariates and Child Developmental Milestones and Socioemotional Problems and Competencies **eTable 3.** Associations Between Vitamin D Supplementation (1200-IU vs. 400-IU) and Child Developmental Milestone Subscale Scores **eTable 4.** Associations Between Vitamin D Supplementation (1200-IU vs. 400-IU) and Social-Emotional Problems and Competencies Subscale Scores **eTable 5.** Associations Between 25(OH)D and Developmental Milestones **eTable 6.** Associations Between 25(OH)D and Child Developmental Milestone Subscale Scores **eTable 7.** Associations Between 25(OH)D and Social-Emotional Problems and Competencies **eTable 8.** Associations Between 25(OH)D and Social-Emotional Problems and Competencies Subscale Scores

Supplement 3.
Data Sharing Statement
