# Supplementary material for: Effect of High-Dose vs Standard-Dose Vitamin D Supplementation on Neurodevelopment of Healthy Term Infants: A Randomized Clinical Trial
Source: JAMA Netw Open. 2021 Sep 8;4(9):e2124493. doi: 10.1001/jamanetworkopen.2021.24493 (PMC8427371; doi:10.1001/jamanetworkopen.2021.24493)
Supplement: Supplement 1. — Trial Protocol [file jamanetwopen-e2124493-s001.pdf]

Supplementary Online Content 1

Vitamin D Intervention in Infants (VIDI) trial

PROTOCOL AND STATISTICAL ANALYSIS PLAN

PROTOCOL

Vitamin D Intervention in Infants (VIDI) is a large randomized trial that aims to evaluate effects of two vitamin D supplemental doses in early childhood on bone strength, infections, immunity, allergy, atopy and asthma, neurologic and cognitive development, and genetic regulation of mineral homeostasis.

This protocol comprises the applied methods to evaluate the effects of vitamin D supplementation on the primary outcomes of the study: developmental milestones and social-emotional problems and competencies in the first two years of life. Methods for additional outcomes have been described in Helve et al, 2017.[1]

Background

Both cutaneously synthesized and vitamin D obtained from diet and supplements contribute to circulating 25-hydroxyvitamin D concentrations. The optimal 25-hydroxyvitamin D concentration is still under discussion. In 2011, Institute of Medicine guidelines stated that 25-hydroxyvitamin D concentrations above 50 nmol/L are required for normal body functions, including linear growth and bone mass accrual.[2] According to the Endocrine Society, concentrations above 75 nmol/L may be necessary to achieve optimal long-term health benefits.[3]

In Finland, Vitamin D supplementation has been recommended to all infants since the 1940's, but in line with the declining prevalence of rickets in our country, the recommended doses have gradually decreased. The present Finnish Nutritional Council guidelines recommend 10 μg (400 IU) of vitamin D3 supplementation daily for all infants from the age of 2 weeks to 2 years. Despite these recommendations, 20 % of 14-month-old children are shown to be vitamin D deficient (< 50 nmol/L).[4]

Normal bone development and growth requires adequate intake of minerals, such as calcium and phosphate. Parathyroid hormone and biologically active form of vitamin D, 1,25-dihydroxyvitamin D, regulate calcium and phosphate concentrations. In addition, 1,25-dihydroxyvitamin D has direct effect on bone cells. Insufficient vitamin D supply results in inadequate bone mineralisation at growth plates and leads to rickets.[5] In addition to its skeletal effects, vitamin D is a steroid hormone with diverse physiological roles in neurological, immune and inflammatory disorders.[6,7] Vitamin D is neuroprotective[6,8] and takes part in regulating the development, differentiation and axonal ramification of nerve cells through neurotrophic factors, and in gene regulation. Nuclear receptors of vitamin D and the presence of specific enzymes that regulate its intracellular conversion to active form have been identified in different parts of the brain.[9] Vitamin D may be important for brain development especially in the early years of life when the brain is developing rapidly and is sensitive to nutrient deficiencies.[10]

Lower vitamin D concentrations are associated with neurodevelopmental disorders, including autism spectrum disorder (ASD) and attention-deficient hyperactivity disorder (ADHD) in children.[11,12] Associations between vitamin D concentrations and cognitive and motor functioning has not been systematically found.[13] However, previous evidence is based on observational studies and no causal relationship can be inferred. Randomized controlled trials (RCT),[14–17] as well as non-randomized trials,[18–22] on vitamin D supplementation in children have been small-scale studies, focused typically on symptom severity among children with ASD or ADHD, and reported mixed findings. Further studies are needed to test whether vitamin D supplementation from infancy on in healthy community-based children provides benefits on neurodevelopment.

Primary outcomes

Primary outcomes of the study are developmental milestones measured with the Ages and Stages Questionnaire (ASQ) at age 12 and 24 months and social-emotional problems and competencies measured with Infant-Toddler Social Emotional Assessment (ITSEA) at age 24 months.

Participants and methods

A total of 1 000 families are recruited and informed consent is obtained 1-2 days after the delivery at the Kätilöopisto Maternity Hospital in Helsinki. We include white northern European women with a singleton pregnancy and without regular medication. Healthy infants born at term (37-42 weeks) and with weight appropriate for gestational age are included in the study. Exclusion criteria for the infants are: seizures, need for early antibiotic treatment, need for nasal continuous positive airway pressure > 24 hours, extended phototherapy > 72 hours, need for nasogastric tube > 24 hours or intravenous glucose infusion. Data on family background (parents' socio-economic status, health, lifestyle factors) and maternal dietary status are documented with a questionnaire. At birth, a cord blood sample (20 ml) is taken and stored for later analyses.

Participating infants are randomised to receive either the currently recommended vitamin D3 supplementation of 10 μg (400 IU) daily or a higher dose of 30 μg (1200 IU) daily from age 2 weeks to 2 years. Boys and girls are randomised separately in blocks of 50. Randomisation is performed by the Helsinki University Hospital Pharmacy. The study is double-blinded. Vitamin D is administered orally as vitamin D3, with a dose of 5 drops a day for both concentrations. The families record daily vitamin D supplementation.

Follow-up

Participants are assessed at a study outpatient clinic by a study nurse and/or pediatrician at 12 months and 24 months of age. Data on developmental milestones are collected with Ages and Stages Questionnaire (ASQ)[23,24] at 12- and 24-month and data on social-emotional problems and competencies with Infant-Toddler Social Emotional Assessment (ITSEA)[25] at 24-month follow-ups. Blood samples are taken for biochemistry. Families are provided study diaries where they keep daily records on dosing of vitamin D3 supplement.

Specific methods

Biochemical markers

Serum 25-hydroxyvitamin D concentration is measured from serum samples with an automated IDSiSYS analyser (IDS Ltd., Bolton, UK) which employs a chemiluminescence immunoassay (CLIA) with high sensitivity, a fast protocol, with a 10-μl specimen volume. The method is validated against LC-MS in-house as well as by the manufacturer. Analyses are performed at the Pediatric Research Centre laboratory in Biomedicum, University of Helsinki (Helsinki, Finland). Reproducibility is ensured by adhering to the Vitamin D External Quality Assessment Scheme (DEQAS, Charing Cross Hospital, London UK). The IDS-iSYS immunoassay will also be used to analyse serum intact parathyroid hormone concentration from serum samples.

Ionised calcium (adjusted to pH 7.40, normal range 1.16-1.39 and 1.17-1.35 mmol/L for age groups 1- 12 months and 24 months respectively) is analysed from serum samples at 12 and 24 months at the Central Laboratory of Helsinki University Hospital (HUSLAB) using ABL 90 FLEX or ABL

835 FLEX blood gas analysers. HUSLAB is an accredited laboratory adhering to international (T055) SFS-EN ISO 15189 and SFS-EN ISO/IEC 17025 standards.

Developmental milestones

Developmental milestones are assessed with parent-reported Ages and Stages Questionnaire (ASQ) 3rd edition. ASQ is a reliable and valid tool with high sensitivity and specificity for screening children requiring further developmental assessment.[23,24] It comprises 21 age-specific questionnaires, covering 1 through 66 months of age, each with six items in each of five developmental domains: communication, gross motor, fine motor, problem solving, and personal/social (solitary social play and play with toys and other children) skills.[23] Subscale scores range from 0 to 60. We chose the 12-month (11-13 months) and 24-month (23-25.5 months) questionnaires.

Social-emotional problems and competencies

Social-emotional problems and competencies are assessed with parent-reported Infant-Toddler Social Emotional Assessment (ITSEA). It is an adult-report questionnaire for 12-to 36-month olds.[25] It includes 169 items containing a statement about the child's behavior during the last month. The scale has good psychometric properties.[25]

Ethical issues and research permits

A recent intervention study confirmed the safety of vitamin D supplementation in infants with a daily dose of 50 µg (2000 IU) daily.[26] Furthermore, we conducted a pilot study in 113 healthy newborns in order to evaluate short-term effects and safety of 3 different vitamin D3 doses (10 µg, 30 µg, 40 µg daily).[27] No adverse events occurred and all doses were deemed safe. Based on the results, we chose daily doses of 10 µg and 30 µg for the intervention study.

An external clinical research institute monitors the study and possible adverse effects. As a safety protocol, the infants are monitored for hypercalcemia at follow-up visits. If the calcium concentration exceeds the upper reference limit of ionised calcium by ≥10%, defined as ionised calcium concentration above 1.53 mmol/L at 6 and 12 months and 1.48 mmol/L at 2 years follow-up, the ionised calcium and 25-hydroxyvitamin D concentrations will be repeatedly measured, symptoms indicative of hypercalcemia will be evaluated, and, if necessary, dosing of vitamin D supplementation adjusted.

Blood samples are taken as follows: A) 20 ml from the umbilical vein after cord clamping at birth, B) 15 ml at age 1 year, and C) ≤ 20 ml at age 2 years. These volumes are clearly below the allowed maximal volumes for research sampling (approximate limits for research purposes are for ages 1 year: 24 ml and 2 years: 36 ml; i.e. 3% of circulating blood volume).

The radiation exposure from pQCT measurements is estimated to be 30 µSv, and exposure from whole-body DXA 50 µSv. This total dose of approximately 80 µSv equals radiation exposure during an overseas flight or 2 weeks' background radiation, and can thus be regarded as insignificant.[28] Similar methods have previously been used to study bone variables in newborns.[27] The vitamin D3 supplements are provided by Orion Pharmaceuticals free of charge. The study is researcher initiated and independent.

Informed consent is obtained from the parents at recruitment. An ethical approval has been obtained from the Research Ethics Committee of the Hospital District of Helsinki and Uusimaa (ID 107/13/03/03/2012) including permission to keep a research register of collected data where the anonymity of all participants is secured with an identification number. Research permits from Children's Hospital are valid until 2018. The project protocol is registered into ClinicalTrial.com (NCT01723852).

STATISTICAL ANALYSIS PLAN

Sample size calculation and statistical analyses

We aim at detecting a 0.2 SD unit difference between continuous variables (developmental milestones and social-behavioral problems and competencies) and Odds Ratios >2.0 in categorical variables (mild developmental delay, clinically possible significant problems).[29] In order to reach statistical power of 80% with significance level of 0.05, a total of 199 and 572 participants, respectively, are necessary. The trial was designed with a planned sample of 1000 study participants. We estimate a possible drop-out rate of 20% for each follow-up.

Statistical methods

Standard statistical methods such as linear, logistic and Tobit and moderation analytic strategies with R/SPSS/MPlus will be used to study the impact of vitamin D supplementation and/or 25(OH)D concentrations, and relevant covariates, on main outcomes.

If needed, logarithmic transformation for nonnormal variables is performed in order to achieve normal distribution. Main outcome measures are analysed according to intention-to-treat and protocol-based manner.

To account for multiple testing and control for the false detection rate (FDR), *p*-values from the analysis within primary/secondary analysis were used to derive an FDR-adjusted *p*-values with a FDR-procedure setting the false discovery rate at 0.05.[30]

References

1. Helve O, Viljakainen H, Holmlund-Suila E, et al. Towards evidence-based vitamin D supplementation in infants: Vitamin D intervention in infants (VIDI) - study design and methods of a randomised controlled double-blinded intervention study. *BMC Pediatr*. 2017;17. doi:10.1186/s12887-017-0845-5
2. Ross AC. The 2011 report on dietary reference intakes for calcium and vitamin D. *Public Health Nutr*. 2011;14:938-939. doi:10.1017/S1368980011000565
3. Holick MF, Binkley NC, Bischoff-Ferrari HA, et al. Guidelines for preventing and treating vitamin D deficiency and insufficiency revisited. *J Clin Endocrinol Metab*. 2012;97:1153-1158. doi:10.1210/jc.2011-2601
4. Viljakainen HT, Korhonen T, Hytinantti T, et al. Maternal vitamin D status affects bone growth in early childhood-a prospective cohort study. *Osteoporos Int*. 2011;22:883-891. doi:10.1007/s00198-010-1499-4
5. Elder CJ, Bishop NJ. Rickets. *Lancet*. 2014;383:1665-1676. doi:10.1016/S0140-6736(13)61650-5
6. Eyles DW, Feron F, Cui X, et al. Developmental vitamin D deficiency causes abnormal brain

development. *Psychoneuroendocrinology*. 2009;34:S247-S257. doi:10.1016/j.psyneuen.2009.04.015

7. Eyles DW, Burne THJ, McGrath JJ. Vitamin D, effects on brain development, adult brain function and the links between low levels of vitamin D and neuropsychiatric disease. *Front Neuroendocrinol*. 2013;34:47-64. doi:10.1016/j.yfrne.2012.07.001
8. Wrzosek M, Lukaszkiewicz J, Wrzosek M, et al. Vitamin D and the central nervous system. *Pharmacol Reports*. 2013;65:271-278. doi:10.1016/S1734-1140(13)71003-X
9. Eyles DW, Smith S, Kinobe R, Hewison M, McGrath JJ. Distribution of the Vitamin D receptor and 1α-hydroxylase in human brain. *J Chem Neuroanat*. 2005;29:21-30.
10. Schwarzenberg SJ, Georgieff MK, Committee on Nutrition. Advocacy for improving nutrition in the first 1000 days to support childhood development and adult health. *Pediatrics*. 2018;141:pii: e20173716. doi:10.1542/peds.2017-3716
11. Föcker M, Antel J, Ring S, et al. Vitamin D and mental health in children and adolescents. *Eur Child Adolesc Psychiatry*. 2017;26:1043-1066. doi:10.1007/s00787-017-0949-3
12. Khoshbakht Y, Bidaki R, Salehi-Abargouei A. Vitamin D status and attention deficit hyperactivity disorder: A systematic review and meta-analysis of observational studies. *Adv Nutr*. 2018;9:9-20. doi:10.1093/advances/nmx002
13. Mutua AM, Mogire RM, Elliott AM, et al. Effects of vitamin D deficiency on neurobehavioural outcomes in children: A systematic review. *Wellcome Open Res*. 2020;5:28. doi:10.12688/wellcomeopenres.15730.1
14. Mohammadpour N, Jazayeri S, Tehrani-Doost M, et al. Effect of vitamin D supplementation as adjunctive therapy to methylphenidate on ADHD symptoms: A randomized, double blind, placebo-controlled trial. *Nutr Neurosci*. 2018. doi:10.1080/1028415X.2016.1262097
15. Saad K, Abdel-Rahman AA, Elserogy YM, et al. Randomized controlled trial of vitamin D supplementation in children with autism spectrum disorder. *J Child Psychol Psychiatry Allied Discip*. 2018. doi:10.1111/jcpp.12652
16. Kerley CP, Power C, Gallagher L, Coghlan D. Lack of effect of Vitamin D 3 supplementation in autism: A 20-week, placebo-controlled RCT. *Arch Dis Child*. 2017. doi:10.1136/archdischild-2017-312783
17. Azzam HME, Sayyah H, Youssef S, et al. Autism and vitamin D: An intervention study. *Middle East Curr Psychiatry*. 2015. doi:10.1097/01.XME.0000457269.05570.78
18. Elshorbagy HH, Barseem NF, Abdelghani WE, et al. Impact of Vitamin D Supplementation on Attention-Deficit Hyperactivity Disorder in Children. *Ann Pharmacother*. 2018. doi:10.1177/1060028018759471
19. Saad K, Abdel-rahman AA, Elserogy YM, et al. Vitamin D status in autism spectrum disorders and the efficacy of vitamin D supplementation in autistic children. *Nutr Neurosci*. 2016. doi:10.1179/1476830515Y.0000000019
20. Feng J, Shan L, Du L, et al. Clinical improvement following vitamin D3 supplementation in Autism Spectrum Disorder. *Nutr Neurosci*. 2017. doi:10.1080/1028415X.2015.1123847
21. Ucuz II, Dursun OB, Esin IS, et al. The relationship between Vitamin D, autistic spectrum disorders, and cognitive development: Do glial cell line-derived neurotrophic factor and nerve growth factor play a role in this relationship? *Int J Dev Disabil*. 2015;6:222-230. doi:10.1179/2047387714Y.0000000054
22. Jia F, Wang B, Shan L, Xu Z, Staal WG, Du L. Core symptoms of autism improved after vitamin D supplementation. *Pediatrics*. 2015;135:e196-e198. doi:10.1542/peds.2014-2121
23. Squires J, Bricker D. *Ages and Stages Questionnaire (ASQ): A Parent Completed Child Monitoring System (3rd Ed.)*. Baltimore: MD: Brooks Publishing Company; 2009.
24. Squires J, Bricker D, Potter L. Revision of a parent-completed developmental screening tool: Ages and stages questionnaires. *J Pediatr Psychol*. 1997;22(3):313-328. doi:10.1093/jpepsy/22.3.313

25. Briggs-Gowan M, Carter AS. Preliminary acceptability and psychometrics of the Infant–Toddler Social and Emotional Assessment (ITSEA): A new adult-report questionnaire. *Infant Ment Health J*. 1998;19:422-445.
26. Wicklow BA, Taback SP. Feasibility of a type 1 diabetes primary prevention trial using 2000 IU vitamin D3 in infants from the general population with increased HLA-associated risk. *Ann N Y Acad Sci*. 2006;1079:310-312. doi:10.1196/annals.1375.047
27. Holmlund-Suila E, Viljakainen H, Hytinantti T, Lamberg-Allardt C, Andersson S, Mäkitie O. High-dose vitamin D intervention in infants - Effects on vitamin D status, calcium homeostasis, and bone strength. *J Clin Endocrinol Metab*. 2012;97:4139-4147. doi:10.1210/jc.2012-1575
28. Blake GM, Naeem M, Boutros M. Comparison of effective dose to children and adults from dual X-ray absorptiometry examinations. *Bone*. 2006;38:935-942. doi:10.1016/j.bone.2005.11.007
29. Chen H, Cohen P, Chen S. How big is a big odds ratio? Interpreting the magnitudes of odds ratios in epidemiological studies. *Commun Stat Simul Comput*. 2010;39:860-864. doi:10.1080/03610911003650383
30. Benjamini Y, Hochberg Y. Controlling the false discovery rate: a practical and powerful approach to multiple testing. *J R Stat Soc*. 1995;57:289-300.
