# Supplementary material for: Effect of High-Dose vs Standard-Dose Vitamin D Supplementation on Neurodevelopment of Healthy Term Infants: A Randomized Clinical Trial
Source: JAMA Netw Open. 2021 Sep 8;4(9):e2124493. doi: 10.1001/jamanetworkopen.2021.24493 (PMC8427371; doi:10.1001/jamanetworkopen.2021.24493)
Supplement: Supplement 2. — eAppendix 1. Supplementary Methods eReferences eAppendix 2. The Report From Immunodiagnostic Systems Containing the Linear Regression Equation for Correction of Cord Blood 25-Hydroxyvitamin D Concentration eTable 1. Comparison of Analytic Sample Against Cohort Members Who Could Not Be Included Due to Missing Data (Attrition Group) eTable 2. Associations Between Covariates and Child Developmental Milestones and Socioemotional Problems and Competencies eTable 3. Associations Between Vitamin D Supplementation (1200-IU vs. 400-IU) and Child Developmental Milestone Subscale Scores eTable 4. Associations Between Vitamin D Supplementation (1200-IU vs. 400-IU) and Social-Emotional Problems and Competencies Subscale Scores eTable 5. Associations Between 25(OH)D and Developmental Milestones eTable 6. Associations Between 25(OH)D and Child Developmental Milestone Subscale Scores eTable 7. Associations Between 25(OH)D and Social-Emotional Problems and Competencies eTable 8. Associations Between 25(OH)D and Social-Emotional Problems and Competencies Subscale Scores [file jamanetwopen-e2124493-s002.pdf]

# Supplementary Online Content

Tuovinen S, Räikkönen K, Holmlund-Suila E, et al. Effect of high-dose vs standard-dose vitamin D supplementation on neurodevelopment of healthy term infants: a randomized clinical trial. *JAMA Netw Open.* 2021;4(9):e2124493.
doi:10.1001/jamanetworkopen.2021.24493

**eAppendix 1.** Supplementary Methods
**eReferences**
**eAppendix 2.** The Report From Immunodiagnostic Systems Containing the Linear Regression Equation for Correction of Cord Blood 25-Hydroxyvitamin D Concentration
**eTable 1.** Comparison of Analytic Sample Against Cohort Members Who Could Not Be Included Due to Missing Data (Attrition Group)
**eTable 2.** Associations Between Covariates and Child Developmental Milestones and Socioemotional Problems and Competencies
**eTable 3.** Associations Between Vitamin D Supplementation (1200-IU vs. 400-IU) and Child Developmental Milestone Subscale Scores
**eTable 4.** Associations Between Vitamin D Supplementation (1200-IU vs. 400-IU) and Social-Emotional Problems and Competencies Subscale Scores
**eTable 5.** Associations Between 25(OH)D and Developmental Milestones
**eTable 6.** Associations Between 25(OH)D and Child Developmental Milestone Subscale Scores
**eTable 7.** Associations Between 25(OH)D and Social-Emotional Problems and Competencies
**eTable 8.** Associations Between 25(OH)D and Social-Emotional Problems and Competencies Subscale Scores

This supplementary material has been provided by the authors to give readers additional information about their work.

© 2021 Tuovinen S et al. *JAMA Network Open.*

**eAppendix 1.** Supplementary Methods

## Family background data

We collected family demographics, including data on health and lifestyle factors, with self-administered research questionnaires at recruitment. Data on gestation and delivery, and on infant demographics, came from electronic hospital records. Season of birth was categorized as in winter (December, January, February), spring (March, April, May), summer (June, July, August) or autumn (September, October, November). Mothers' educational level from the questionnaires was graded from 1 (=comprehensive school) to 6 (university degree) and then recategorized into 2 levels: low educational level (=less than a bachelor's degree) and high educational level (=at least a bachelor's degree). Data also included maternal smoking before pregnancy and after the delivery collected with self-administered research questionnaires and duration of breastfeeding determined based on prospectively collected study diaries.

## Laboratory analyses

25-hydroxyvitamin D concentration was analyzed with an IDS-iSYS fully automated immunoassay system with chemiluminescense detection (Immunodiagnostic Systems Ltd., Bolton, UK). Cord plasma 25-hydroxyvitamin D concentrations were corrected with an equation (19.13 + 0.897 * cord plasma 25-hydroxyvitamin D value) to be comparable with serum 25-hydroxyvitamin D concentrations. The equation was based on comparison of 25- hydroxyvitamin D measurements in samples from 84 study subjects for whom both cord plasma and serum samples were available. In addition, because of manufacturer's changes in the IDS-iSYS system between 2014 and 2016, the cord serum 25-hydroxyvitamin D concentrations were corrected by a linear regression equation (correct value (nmol/L) © 2018 American Medical Association. All rights reserved. 3 = [(initial value) – 8.2] / 0.99), provided by the manufacturer (see eAppendix 2). We re-analyzed a subsample of 77 samples and verified the correction (adjusted correlation coefficient = 0.922, standard error of the estimate = 9.2 nmol/L).

All samples were analyzed between 2014 and 2016 with intra-assay variation <13% for cord blood and <5% for 12- and 24-month samples. Accuracy of the serum 25-hydroxyvitamin D analysis was monitored by continued participation in the vitamin D External Quality Assessment Scheme (DEQAS, Charing Cross Hospital, London, UK). This method showed a constant <8% positive bias based on the NIST (National Institute of Standards and Technology) Reference Measurement Procedure during 2014 and 2016.

The IDS-iSYS immunoassay also served to analyze intact parathyroid hormone (PTH) from 12- and 24-month serum samples. The reference range for PTH was 11.5-78.4 ng/L; the lowest detection limit was 4.5 ng/L. PTH values less than that were coded as 4.4 ng/L.

Plasma ionized calcium, adjusted to pH 7.40, was analyzed from capillary samples at 6 months and from serum samples at 12 and 24 months at the Central Laboratory of Helsinki University Hospital (HUSLAB) with the blood gas analyzer ABL 90 FLEX or ABL 835 FLEX. The reference range for ionized calcium at 6 and 12 months was 1.16-1.39 mmol/L and at 24 months 1.17-1.35 mmol/L.

© 2021 Tuovinen S et al. *JAMA Network Open.*

## Outcome measures

Developmental milestones

The Ages and Stages Questionnaires (ASQ) 3rd edition[1,2] was translated into Finnish, back-translated and approved by the publisher. It is a tool with good test-retest reliability, intra-observer reliability, internal consistency and validity and high sensitivity and specificity to screen children at risk of a delay and requiring further developmental assessment.[2,3]

It consists of 19 different questionnaires covering the age-range of 4 to 60 months, and each takes 10–15 min to complete. The questionnaires cover five different subscales or domains: communication, gross motor, fine motor, problem solving and personal social skills. Each of the five domain is assessed by six questions on developmental mile-stones. Items are scored “10” if the child can master the skill, “5” if the skill is emerging/occasional, and “0” if the child cannot master the skill. On each of these domains, the scores range from 0 to 60 with the highest value indicating that the child can master the skill.

According to the ASQ manual and validation studies[2–4] cut-off point ≤ 2 standard deviations (SD) below the mean on any domain is considered to indicate developmental delay. Scores between -2 SDs and -1 SD on any domain were considered to indicate mild developmental delay. Children with scores 1 SD above of the mean were considered to be developing typically for child’s age.[2] A cut-off point of ≤-1 SD for the total ASQ score was considered the best for screening purposes to identify those at risk of a delay and to indicate mild developmental delay[4] and was chosen for the current study.

Social-emotional problems and competencies

Infant-Toddler Social Emotional Assessment (ITSEA) is a tool to assess social-emotional problems and competencies of children 12 to 36 months of age.[5] It includes 169 items containing a statement about the child’s behavior during the last month. According to the manual and validation studies the scale has good psychometric properties; test-retest reliability, interrater reliability, internal consistency, and construct validity.[5–9]

The questionnaire covers four core domains: Externalizing (24 items; measuring activity, impulsivity, aggression and defiance), Internalizing (32 items; measuring depression, withdrawal, anxiety, separation distress, and inhibition), Dysregulation (34 items; measuring problems in sleeping, eating, emotional reactivity and regulation, and unusual sensory sensitivities), and Competence domain (37 items; measuring compliance, attention regulation, imitation and pretend play skills, mastery motivation, empathy, emotional awareness, and prosocial peer behaviors). It includes 17 specific problems and competencies subscales (described under domains).[5] The respondent rates each item on a 3 point scale (0=not true/rarely, 1=somewhat true/sometimes, 2=very true/always).

ITSEA domain scores can be reported as T-scores with a mean of 50 and a SD of 10. Problem domain scores (i.e., Externalizing, Internalizing, Dysregulation) of 65 or higher (i.e., ≥1.5 SD above the mean) and Competence domain scores of 35 or lower (i.e., ≥1.5 SD below the mean) are considered indicative of a deficit or delay (i.e., “clinical concern”) in previous studies.[7,10,11]

© 2021 Tuovinen S et al. *JAMA Network Open.*

**eReferences**


1. Squires J, Bricker D. *Ages and Stages Questionnaire (ASQ): A Parent Completed Child Monitoring System (3rd Ed.)*. Baltimore: MD: Brooks Publishing Company; 2009.
2. Squires J, Bricker D, Potter L. Revision of a parent-completed developmental screening tool: Ages and stages questionnaires. *J Pediatr Psychol*. 1997;22(3):313-328. doi:10.1093/jpepsy/22.3.313
3. Kerstjens JM, Bos AF, ten Vergert EMJ, de Meer G, Butcher PR, Reijneveld SA. Support for the global feasibility of the Ages and Stages Questionnaire as developmental screener. *Early Hum Dev*. 2009;85:443-447. doi:10.1016/j.earlhumdev.2009.03.001
4. Steenis LJP, Verhoeven M, Hessen DJ, van Baar AL. Parental and professional assessment of early child development: The ASQ-3 and the Bayley-III-NL. *Early Hum Dev*. 2015. doi:10.1016/j.earlhumdev.2015.01.008
5. Briggs-Gowan M, Carter AS. Preliminary acceptability and psychometrics of the Infant–Toddler Social and Emotional Assessment (ITSEA): A new adult-report questionnaire. *Infant Ment Health J*. 1998;19:422-445.
6. Carter AS, Briggs-Gowan MJ, Jones SM, Little TD. The Infant-Toddler Social and Emotional Assessment (ITSEA): Factor structure, reliability, and validity. *J Abnorm Child Psychol*. 2003;31:495-514. doi:10.1023/A:1025449031360
7. Carter AS, Briggs-Gowan MJ. *Infant-Toddler Social and Emotional Assessment Examiners Manual*. San Antonio, TX: PsychCorp: Harcourt Assessment; 2006.
8. Halle TG, Darling-Churchill KE. Review of measures of social and emotional development. *J Appl Dev Psychol*. 2016;45:8-18. doi:10.1016/j.appdev.2016.02.003
9. Sanner N, Smith L, Wenzel-Larsen T, Moe V. Early identification of social-emotional problems: Applicability of the Infant-Toddler Social Emotional Assessment (ITSEA) at its lower age limit. *Infant Behav Dev*. 2016;42:69-85. doi:10.1016/j.infbeh.2015.11.001
10. van Ewijk R, Huibers MHW, Manshande ME, et al. Neurologic sequelae of severe chikungunya infection in the first 6 months of life: a prospective cohort study 24-months post-infection. *BMC Infect Dis*. 2021;21:179. doi:10.1186/s12879-021-05876-4
11. Thurm A, Manwaring SS, Cardozo Jimenez C, et al. Social-emotional and behavioral problems in toddlers with language delay. *Infant Ment Health J*. 2018:569-580. doi:10.1002/imhj.21735


© 2021 Tuovinen S et al. *JAMA Network Open.*

**eAppendix 2.** The Report From Immunodiagnostic Systems Containing the Linear Regression Equation for Correction of Cord Blood 25-Hydroxyvitamin D Concentration

© 2021 Tuovinen S et al. *JAMA Network Open.*

immunodiagnosticsystems

October 23th, 2014

RE: DEQAS July 2014 25-hydroxyvitamin D positive bias

Dear Valued Customer,

The 25-Hydroxyvitamin DEQAS - July 2014 distribution report indicates that the results obtained with the IDS-iSYS 25-Hydroxy Vitamin $D^S$ assay were outside the ± 25% criteria.

Immunodiagnostic Systems launched an internal investigation by verifying the alignment between the IDS-iSYS 25-Hydroxy Vitamin $D^S$ and the ID-LC-/MS/MS 25(OH)D Reference Method Procedure (RMP) using the single donor serum samples from the Vitamin D Standardization Program (VDSP). The DEQAS July 2014 distribution and our internal serum samples panels were also measured in multiple reagent lots and systems.

We have confirmed a bias in DEQAS - July 2014 distribution. The regression slope between the IDS-iSYS assay and the RMP is slightly higher than previously communicated in the Product Notification NIS2700S/04 (1.06 vs. 1.04); the mean % bias is 12% versus -2%. The bias occurred due to the implemementing of a new internal serum panel preparation with target value slightly higher than the LC-MS/MS value.

From kit lot 2191 and onward, we have adjusted the internal serum panel target value to correct the bias. The summary of IDS-iSYS 25-Hydroxy Vitamin $D^S$ Traceability is enclosed for your references. The results confirm the alignment against the ID-LC-/MS/MS 25(OH)D Reference Method Procedure (RMP).

Immunodiagnostic Systems strives to provide you with products of the highest quality. We value your business and thank you for your continued support. Please contact your local IDS representative if you have any further questions regarding this information.

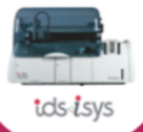





© 2021 Tuovinen S et al. *JAMA Network Open.*

## IDS-iSYS 25-Hydroxy Vitamin D$^{S}$ (ng/mL) VDSP Traceability

The single donor serum samples (n = 70) with RMP ID-LC-MS/MS 25(OH)D target value ranging from 9.0 – 79.2 ng/mL from the Vitamin D Standardization Program (VDSP) were used to verify the IDS-iSYS alignment in May 2014. The same samples were measured in September 2014 to confirm the assay traceability. From kit lot 2191 onward, we have adjusted the internal serum panel target value to correct the bias.

| | Passing-Bablok regression | Linear regression | Statistical summary |
|---|---|---|---|
| **Product Notification NIS2700S/04 May 2014** | 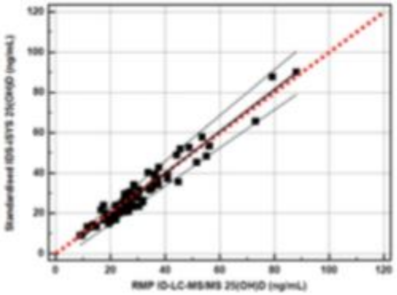 | 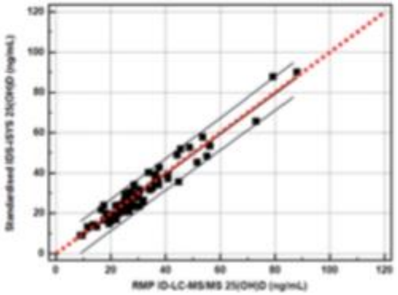 | The Passing-Bablok regression between the IDS-iSYS (y) and the RMP ID-LC-MS/MS (x) is:<br>IDS-iSYS = 1.04 x (RMP) -1.6 ng/mL<br>95 % CI. slope: 0.95 to 1.13<br>95 % CI. intercept: -4.2 to 0.5 ng/mL<br>Pearson corr. coeff. r: 0.967 (0.948 to 0.980), P<0.0001<br>Mean %bias: -2.0%<br>The linear regression equation is:<br>IDS-iSYS = 1.00 x (RMP) – 0.8 ng/mL<br>95 % CI. slope: 0.94 to 1.07<br>95 % CI. intercept: -3.0 to 1.4 ng/mL |
| **Alignment verification September 2014** | 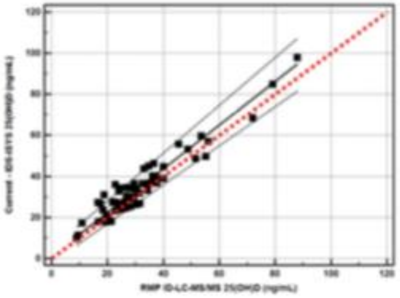 | 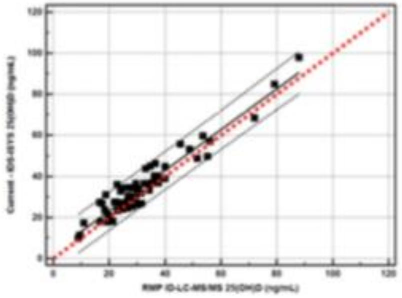 | Although the Passing-Bablok regression yields a slope >1.05, the slope of linear regression is 0.99.<br>The Passing-Bablok regression is:<br>IDS-iSYS = 1.06 x (RMP) +1.0 ng/mL<br>95 % CI. slope: 0.95 to 1.17<br>95 % CI. intercept: -2.2 to 4.6 ng/mL<br>Pearson corr. coeff. r: 0.954 (0.932 to 0.970), P<0.0001<br>Mean %bias: 12.0%<br>The linear regression equation is:<br>IDS-iSYS = 0.99 x (RMP) + 3.3 ng/mL<br>95 % CI. slope: 0.91 to 1.07<br>95 % CI. intercept: 0.6 to 6.0 ng/mL |
| **Corrected - October 2014** | 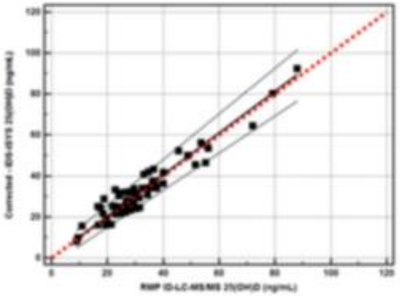 | 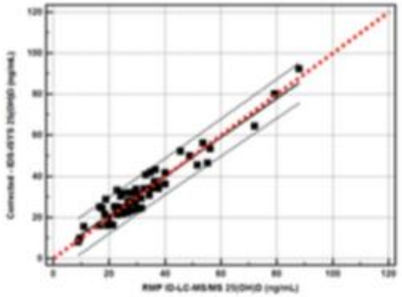 | After the correction, the Passing-Bablok regression yields a slope of 1.02 and the linear regression slope is 0.95. The Passing-Bablok regression is:<br>IDS-iSYS = 1.02 x (RMP) -0.0 ng/mL<br>95 % CI. slope: 0.91 to 1.11<br>95 % CI. intercept: -3.0 to 3.0 ng/mL<br>Pearson corr. coeff. r: 0.954 (0.932 to 0.970), P<0.0001<br>Mean %bias: 3.1%<br>The linear regression equation is:<br>IDS-iSYS = 0.95 x (RMP) + 2.1 ng/mL<br>95 % CI. slope: 0.87 to 1.02<br>95 % CI. intercept: -0.5 to 4.7 ng/mL |

## IDS-iSYS 25-Hydroxy Vitamin D$^{S}$ (nmol/L) VDSP Traceability

The single donor serum samples (n = 70) with RMP ID-LC-MS/MS 25(OH)D target value ranging from 23 – 198 nmol/L from the Vitamin D Standardization Program (VDSP) were used to verify the IDS-iSYS alignment in May 2014. The same samples were measured in September 2014 to confirm the assay traceability. From kit lot 2191 onward, we have adjusted the internal serum panel target value to correct the bias.

| | Passing-Bablok regression | Linear regression | Statistical summary |
|---|---|---|---|
| **Product Notification NIS2700S/04 May 2014** | 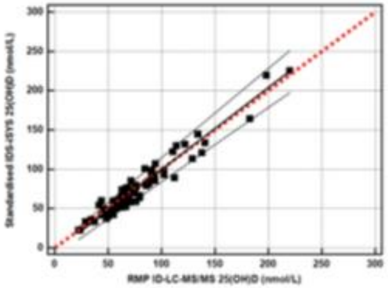 | 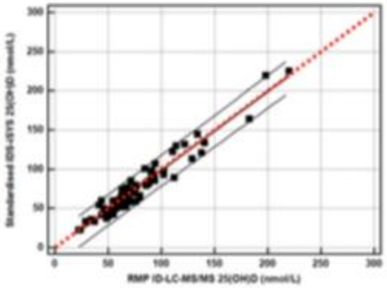 | The Passing-Bablok regression between the IDS-iSYS (y) and the RMP ID-LC-MS/MS (x) is:<br>IDS-iSYS = 1.04 x (RMP) - 4.0 nmol/L<br>95 % CI. slope: 0.95 to 1.13<br>95 % CI. intercept: -10.5 to 1.4 nmol/L<br>Pearson corr. coeff. r: 0.967 (0.948 to 0.980), P<0.0001<br>Mean %bias: -2.0%<br>The linear regression equation is:<br>IDS-iSYS = 1.00 x (RMP) – 2.0 nmol/L<br>95 % CI. slope: 0.94 to 1.07<br>95 % CI. intercept: -7.6 to 3.5 nmol/L |
| **Alignment verification September 2014** | 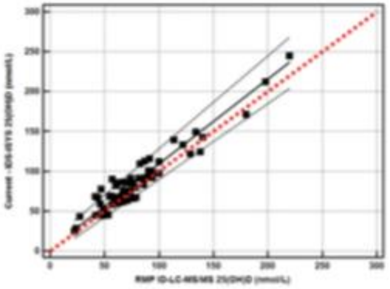 | 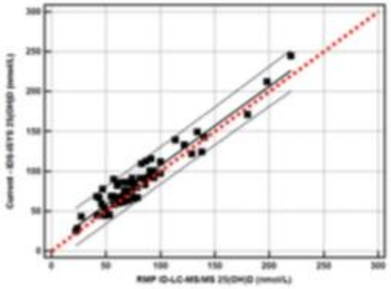 | Although the Passing-Bablok regression yields a slope >1.05, the slope of linear regression is 0.99.<br>The Passing-Bablok regression is:<br>IDS-iSYS = 1.06 x (RMP) +2.6 nmol/L<br>95 % CI. slope: 0.95 to 1.17<br>95 % CI. intercept: -5.4 to 11.5 nmol/L<br>Pearson corr. coeff. r: 0.954 (0.932 to 0.970), P<0.0001<br>Mean %bias: 12.0%<br>The linear regression equation is:<br>IDS-iSYS = 0.99 x (RMP) + 8.2 nmol/L<br>95 % CI. slope: 0.91 to 1.07<br>95 % CI. intercept: 1.5 to 15.0 nmol/L |
| **Corrected - October 2014** | 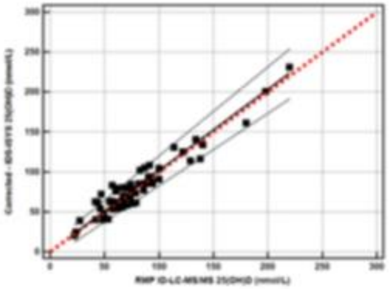 | 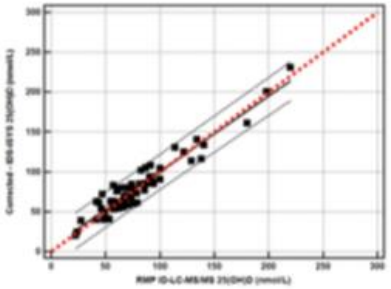 | After the correction, the Passing-Bablok regression yields a slope of 1.02 and the linear regression slope is 0.95. The Passing-Bablok regression is:<br>IDS-iSYS = 1.02 x (RMP) - 0.0 nmol/L<br>95 % CI. slope: 0.91 to 1.11<br>95 % CI. intercept: -7.6 to 8.8 nmol/L<br>Pearson corr. coeff. r: 0.954 (0.932 to 0.970), P<0.0001<br>Mean %bias: 3.1%<br>The linear regression equation is as follow:<br>IDS-iSYS = 0.95 x (RMP) + 2.1 nmol/L<br>95 % CI. slope: 0.87 to 1.02<br>95 % CI. intercept: -0.5 to 4.7 nmol/L |



© 2021 Tuovinen S et al. *JAMA Network Open.*

**eTable 1.** Comparison of Analytic Sample Against Cohort Members Who Could Not Be Included Due to Missing Data (Attrition Group)

| | ASQ 12-month follow-up | | | ASQ 24-month follow-up | | | ITSEA follow-up | | |
|---|---|---|---|---|---|---|---|---|---|
| | **Analytic sample** total N=667 Mean (SD)/ N(%) | **Attrition sample** total N=320 Mean (SD)/ N(%) | **Group difference** (*p*) | **Analytic sample** total N=636 Mean (SD)/ N(%) | **Attrition sample** total N=351 Mean (SD)/ N(%) | **Group difference** (*p*) | **Analytic sample** total N=657 Mean (SD)/ N(%) | **Attrition sample** total N=330 Mean (SD)/ N(%) | **Group difference** (*p*) |
| **Child** | | | | | | | | | |
| Intervention group, 400 IU | 342 (51.3%) | 153 (47.8%) | 0.31 | 324 (50.9%) | 312 (49.1%) | 0.50 | 327 (49.8%) | 168 (50.9%) | 0.74 |
| **At birth** | | | | | | | | | |
| Sex, female | 338 (50.7%) | 154 (48.0%) | 0.45 | 318 (50.0%) | 174 (49.6%) | 0.90 | 331 (50.4%) | 161 (48.8%) | 0.64 |
| Length of gestation, days | 281.1 (7.6) | 281.6 (7.7) | 0.40 | 281.3 (7.5) | 281.3 (7.9) | 0.89 | 281.0 (7.6) | 282.0 (7.7) | 0.052 |
| Season of birth | | | 0.27 | | | 0.06 | | | 0.72 |
| Winter | 128 (19.2%) | 65 (20.3%) | | 121 (19.0%) | 72 (20.5%) | | 129 (19.6%) | 64 (19.4%) | |
| Spring | 283 (42.2%) | 120 (37.5%) | | 275 (43.2%) | 128 (36.5%) | | 264 (40.2%) | 139 (42.1%) | |
| Summer | 138 (20.7%) | 82 (25.6%) | | 127 (20.0%) | 93 (26.5%) | | 144 (21.9%) | 76 (23.0%) | |
| Autumn | 118 (17.7%) | 53 (16.6%) | | 113 (17.8%) | 58 (16.5%) | | 120 (18.3%) | 51 (15.5%) | |
| | | | | | | | | | |
| | | | | | | | | | |

© 2021 Tuovinen S et al. *JAMA Network Open.*

| | ASQ 12-month follow-up | | | ASQ 24-month follow-up | | | ITSEA follow-up | | |
|---|---|---|---|---|---|---|---|---|---|
| | **Analytic sample** total N=667 Mean (SD)/ N(%) | **Attrition sample** total N=320 Mean (SD)/ N(%) | **Group difference** (*p*) | **Analytic sample** total N=636 Mean (SD)/ N(%) | **Attrition sample** total N=351 Mean (SD)/ N(%) | **Group difference** (*p*) | **Analytic sample** total N=657 Mean (SD)/ N(%) | **Attrition sample** total N=330 Mean (SD)/ N(%) | **Group difference** (*p*) |
| 25(OH)D concentration at birth (cord blood, ng/ml) | 82.4 (25.7) | 80.1 (26.2) | 0.18 | 82.7 (26.2) | 79.9 (24.7) | 0.10 | *83.0 (25.3)* | *79.1 (26.8)* | *0.03* |
| Breastfed, months | 10.7 (5.5) | 10.7 (5.8) | 0.91 | 10.8 (5.6) | 10.3 (5.5) | 0.19 | *10.9 (5.5)* | *9.8 (5.5)* | *0.01* |
| **Mother** | | | | | | | | | |
| Age | 31.2 (4.3) | 31.3 (4.6) | 0.83 | 31.2 (4.3) | 31.3 (4.7) | 0.81 | 31.3 (4.3) | 30.9 (4.6) | 0.20 |
| Smoking at childbirth | 90 (13.8%) | 43 (18.9%) | 0.06 | 86 (13.8%) | 47 (18.1%) | 0.10 | 90 (13.9%) | 43 (18.3%) | 0.11 |
| Depressive symptoms at childbirth | 11.9 (6.2) | 12.7 (7.1) | 0.15 | 11.9 (6.3) | 12.6 (6.8) | 0.19 | 11.9 (6.0) | 12.6 (7.4) | 0.17 |
| Educational level, high | *502 (77.5%)* | *159 (70.0%)* | *0.03* | *482 (78.2%)* | *179 (69.1%)* | *0.004* | *507 (79.0%* | *154 (66.1%)* | *<0.001* |

Significant associations marked in italics.

© 2021 Tuovinen S et al. *JAMA Network Open.*

Abbreviation: SD, standard deviation; MD, mean difference; 25(OH)D, 25-hydroxyvitamin D

© 2021 Tuovinen S et al. *JAMA Network Open*.

| **eTable 2.** Associations Between Covariates and Child Developmental Milestones and Socioemotional Problems and Competencies | | | | | | | | | | | | |
|---|---|---|---|---|---|---|---|---|---|---|---|---|
| | **ASQ (SD units)** | | | | **ITSEA (SD units)** | | | | | | | |
| | **Total score at 12-month follow-up** | | **Total score at 24-month follow-up** | | **Externalizing** | | **Internalizing** | | **Dysregulation** | | **Competence** | |
| | **r/MD** | ***p*** | **r/MD** | ***p*** | **r/MD** | ***p*** | **r/MD** | ***p*** | **r/MD** | ***p*** | **r/MD** | ***p*** |
| **Child** | | | | | | | | | | | | |
| **At birth** | | | | | | | | | | | | |
| Sex, male vs. female (ref.) | *-0.21* | *0.008* | *-0.47* | *<0.001* | *0.28* | *<0.001* | -0.02 | 0.78 | 0.03 | 0.67 | *0.30* | *<0.001* |
| Length of gestation, days | *0.09* | *0.02* | 0.03 | 0.52 | 0.03 | 0.45 | 0.02 | 0.61 | 0.05 | 0.21 | -0.02 | 0.64 |
| Season of birth, winter (ref.) | | | | | | | | | | | | |
| Spring | 0.03 | 0.77 | 0.05 | 0.63 | -0.02 | 0.87 | 0.09 | 0.38 | -0.13 | 0.22 | 0.11 | 0.28 |
| Summer | 0.15 | 0.23 | 0.20 | 0.12 | -0.02 | 0.89 | 0.16 | 0.16 | -0.22 | 0.07 | 0.01 | 0.90 |
| Autumn | 0.15 | 0.24 | 0.01 | 0.90 | -0.09 | 0.45 | -0.00 | 0.97 | -0.21 | 0.11 | -0.08 | 0.58 |
| 25(OH)D concentration at birth (cord blood, ng/ml) | 0.02 | 0.59 | 0.02 | 0.55 | -0.02 | 0.67 | -0.01 | 0.83 | -0.01 | 0.84 | -0.01 | 0.7 |
| Breastfed, months | 0.03 | 0.50 | *0.14* | *<0.001* | -0.08 | 0.06 | 0.02 | 0.64 | *0.15* | *<0.001* | 0.03 | 0.51 |
| **At follow-up** | | | | | | | | | | | | |
| Age at corresponding follow-up, months | *0.18* | *<0.001* | *0.09* | *0.02* | 0.05 | 0.25 | 0.07 | 0.09 | *0.10* | *0.01* | 0.05 | 0.19 |
| | | | | | | | | | | | | |

© 2021 Tuovinen S et al. *JAMA Network Open.*

| | **ASQ (SD units)** | | | | **ITSEA (SD units)** | | | | | | | |
|---|---|---|---|---|---|---|---|---|---|---|---|---|
| | **Total score at 12-month follow-up** | | **Total score at 24-month follow-up** | | **Externalizing** | | **Internalizing** | | **Dysregulation** | | **Competence** | |
| | **r/MD** | ***p*** | **r/MD** | ***p*** | **r/MD** | ***p*** | **r/MD** | ***p*** | **r/MD** | ***p*** | **r/MD** | ***p*** |
| **Mother** | | | | | | | | | | | | |
| Age | -0.09 | 0.02 | 0.01 | 0.77 | *-0.11* | *0.005* | -0.05 | 0.25 | 0.03 | 0.42 | -0.03 | 0.46 |
| Smoking at childbirth, yes. vs. no (ref.) | -0.10 | 0.35 | -1.01 | 0.31 | *3.82* | *<0.001* | -0.41 | 0.68 | -0.22 | 0.83 | -0.10 | 0.92 |
| Depressive symptoms at childbirth | -0.06 | 0.13 | -0.07 | 0.10 | *0.23* | *<0.001* | *0.16* | *<0.001* | *0.26* | *<0.001* | *-0.15* | *0.001* |
| Educational level, low vs. high (ref.) | -0.00 | 0.99 | *0.19* | *0.04* | -0.12 | 0.22 | -0.19 | 0.051 | -0.07 | 0.46 | 0.08 | 0.39 |

Child ASQ scores and ITSEA scores are rank-normalized according to Blom's formula and expressed in SD units.

Significant associations marked in italics.

Abbreviation: SD, standard deviation; MD, mean difference; 25(OH)D, 25-hydroxyvitamin D; ASQ, Ages and Stages Questionnaire; ITSEA, Infant-Toddler Social and Emotional Assessment

© 2021 Tuovinen S et al. *JAMA Network Open.*

**eTable 3.** Associations Between Vitamin D Supplementation (1200-IU vs. 400-IU) and Child Developmental Milestone Subscale Scores

| | Model 1 | | Model 2 | |
|---|---|---|---|---|
| **Subscales of developmental milestones** | **B (95% CI)** | ***p*** | **B (95% CI)** | ***p*** |
| **12-month follow-up** | | | | |
| Communication skills | 1.31 (1.00, 1.71) | 0.05 | 1.49 (1.11, 2.02) | 0.01 |
| Gross motor skills | 1.04 (0.79, 1.35) | 0.78 | 1.21 (0.90, 1.63) | 0.21 |
| Fine motor skills | 1.08 (0.82, 1.40) | 0.59 | 1.15 (0.85, 1.54) | 0.37 |
| Problem solving skills | 1.36 (1.03, 1.79) | 0.03 | 1.44 (1.06, 1.95) | 0.02 |
| Personal social skills | 1.02 (0.78, 1.33) | 0.87 | 1.02 (0.76, 1.37) | 0.88 |
| **24-month follow-up** | | | | |
| Communication skills | 1.17 (0.88, 1.56) | 0.27 | 1.16 (0.85, 1.60) | 0.34 |
| Gross motor skills | 1.10 (0.82, 1.48) | 0.53 | 1.17 (0.85, 1.61) | 0.35† |
| Fine motor skills | 1.02 (0.77, 1.35) | 0.87 | 1.09 (0.80, 1.47) | 0.60 |
| Problem solving skills | 0.97 (0.74, 1.27) | 0.81 | 1.04 (0.77, 1.40) | 0.82 |
| Personal social skills | 1.20 (0.91, 1.59) | 0.19 | 1.23 (0.91, 1.67) | 0.18 |

Bs and 95 % CIs from Ordinal logistic regression analyses refer to differences in ASQ developmental milestones subdomain scores of 1200-IU group vs. 4000-IU group (ref.)

Model 1 is crude model.

Model 2 is adjusted for sex, length of gestation, duration of breastfeeding, age at follow-up, maternal age at delivery, maternal smoking and depressive symptoms at childbirth, and maternal educational level (missing values dummy-coded to their own category).

© 2021 Tuovinen S et al. *JAMA Network Open.*

†Adjusted as in Model 2 excluding maternal depressive symptoms. Maternal depressive symptoms were not available for all children which led to a reduced number of participants in analyses and empty or extreme small cells (has no cases) in lower end at the gross motor skills domain.

Abbreviation: B, unstandardized regression coefficient; 95% CI, 95% confidence interval; ASQ, Ages and Stages Questionnaire

© 2021 Tuovinen S et al. *JAMA Network Open.*

**eTable 4.** Associations Between Vitamin D Supplementation (1200-IU vs. 400-IU) and Social-Emotional Problems and Competencies Subscale Scores

| | 400-IU Group | 1200-IU Group | | |
|---|---|---|---|---|
| **Social-emotional problems and competencies subscale scores** | **Mean (SD)** | **Mean (SD)** | **MD (95% CI)** | ***p*** |
| **Externalizing domain** | | | | |
| Activity/Impulsity | | | | |
| Model 1 | -0.03 (0.99) | 0.02 (1.01) | 0.04 (-0.11, 0.20) | 0.59 |
| Model 2 | -0.00 (1.02) | 0.04 (1.03) | 0.06 (-0.11, 0.22) | 0.51 |
| Aggression/defiance | | | | |
| Model 1 | -0.03 (1.02) | 0.03 (0.98) | 0.06 (-0.09, 0.21) | 0.44 |
| Model 2 | -0.06 (1.03) | 0.05 (1.00) | 0.12 (-0.04, 0.28) | 0.14 |
| Peer aggression | | | | |
| Model 1 | -0.06 (0.96) | 0.05 (1.03) | 0.11 (-0.05, 0.26) | 0.17 |
| Model 2 | -0.10 (0.96) | 0.07 (1.03) | 0.16 (-0.00, 0.33) | 0.06 |
| **Internalizing domain** | | | | |
| Depression/Withdrawal | | | | |
| Model 1 | 0.01 (0.99) | -0.01 (1.01) | -0.03 (-0.18, 0.13) | 0.74 |
| Model 2 | 0.03 (1.01) | -0.03 (1.01) | -0.04 (-0.21, 0.13) | 0.62 |
| General anxiety | | | | |
| Model 1 | 0.00 (1.01) | -0.01 (0.99) | -0.01 (-0.16, 0.14) | 0.91 |
| Model 2 | 0.01 (1.04) | -0.00 (0.99) | -0.00 (-0.17, 0.16) | 0.98 |

© 2021 Tuovinen S et al. *JAMA Network Open.*

| Social-emotional problems and competencies subscale scores | 400-IU Group Mean (SD) | 1200-IU Group Mean (SD) | MD (95% CI) | *p* |
|---|---|---|---|---|
| Separation distress | | | | |
| Model 1 | 0.03 (1.01) | -0.03 (0.99) | -0.06 (-0.21, 0.10) | 0.46 |
| Model 2 | 0.04 (1.03) | -0.03 (1.00) | -0.08 (-0.24, 0.09) | 0.36 |
| Inhibition to novelty | | | | |
| Model 1 | -0.01 (1.03) | 0.01 (0.98) | 0.03 (-0.13, 0.18) | 0.75 |
| Model 2 | 0.03 (1.03) | -0.03 (0.98) | -0.05 (-0.22, 0.11) | 0.52 |
| **Dysregulation domain** | | | | |
| Negative emotionality | | | | |
| Model 1 | -0.01 (1.01) | 0.00 (1.00) | 0.01 (-0.14, 0.17) | 0.87 |
| Model 2 | 0.02 (1.03) | -0.00 (1.02) | -0.01 (-0.18, 0.15) | 0.88 |
| Sleep | | | | |
| Model 1 | 0.03 (1.01) | -0.03 (0.99) | -0.06 (-0.22, 0.09) | 0.42 |
| Model 2 | 0.03 (1.01) | -0.03 (0.98) | -0.07 (-0.23, 0.09) | 0.41 |
| Eating | | | | |
| Model 1 | -0.05 (0.99) | 0.05 (1.01) | 0.10 (-0.06, 0.25) | 0.21 |
| Model 2 | -0.04 (1.02) | 0.04 (1.00) | 0.08 (-0.09, 0.25) | 0.35 |
| Sensory sensitivity | | | | |
| Model 1 | -0.06 (1.02) | 0.06 (0.98) | 0.12 (-0.03, 0.28) | 0.12 |
| Model 2 | -0.06 (1.04) | 0.05 (0.98) | 0.12 (-0.05, 0.28) | 0.17 |

© 2021 Tuovinen S et al. *JAMA Network Open.*

| Social-emotional problems and competencies subscale scores | 400-IU Group<br>Mean (SD) | 1200-IU Group<br>Mean (SD) | MD (95% CI) | *p* |
|---|---|---|---|---|
| **Competence domain** | | | | |
| Compliance | | | | |
| Model 1 | 0.04 (0.97) | -0.03 (1.03) | -0.07 (-0.22, 0.09) | 0.38 |
| Model 2 | 0.03 (1.00) | -0.05 (1.02) | -0.08 (-0.24, 0.09) | 0.35 |
| Attention | | | | |
| Model 1 | -0.03 (1.00) | 0.03 (1.01) | 0.06 (-0.09, 0.21) | 0.44 |
| Model 2 | -0.06 (1.01) | 0.03 (1.02) | 0.09 (-0.08, 0.25) | 0.31 |
| Mastery motivation | | | | |
| Model 1 | -0.04 (1.01) | 0.04 (0.99) | 0.08 (-0.08, 0.23) | 0.33 |
| Model 2 | -0.07 (1.02) | 0.03 (1.02) | 0.10 (-0.07, 0.27) | 0.24 |
| Imitation/Play | | | | |
| Model 1 | -0.00 (1.01) | -0.00 (0.99) | -0.00 (-0.16, 0.15) | 0.97 |
| Model 2 | -0.01 (1.00) | 0.01 (1.01) | 0.04 (-0.13, 0.20) | 0.68 |
| Empathy | | | | |
| Model 1 | -0.00 (1.03) | 0.00 (0.97) | 0.00 (-0.15, 0.16) | 0.95 |
| Model 2 | -0.03 (1.04) | 0.02 (0.97) | 0.07 (-0.10, 0.23) | 0.42 |
| Prosocial peer relations | | | | |
| Model 1 | 0.03 (0.94) | -0.02 (1.05) | -0.05 (-0.21, 0.10) | 0.52 |
| Model 2 | 0.03 (0.94) | -0.03 (1.08) | -0.04 (-0.20, 0.13) | 0.66 |

© 2021 Tuovinen S et al. *JAMA Network Open.*

Values represent means in SD units and MDs with 95% CIs in ITSEA subscale scores from linear regression analyses. To facilitate comparison of effect sizes all continuous outcome variables were standardized to the mean of 0 and SD of 1.

Model 1 is crude model.

Model 2 is adjusted for sex, length of gestation, duration of breastfeeding, age at follow-up (missing values replaced with a mean of the sample), maternal age at delivery, maternal smoking and depressive symptoms at childbirth, and maternal educational level (missing values dummy-coded to their own category).

Abbreviation: MD, mean difference; SD, standard deviation; 95% CI, 95% Confidence Interval; SD, standard deviation; ITSEA, Infant-Toddler Social and Emotional Assessment

© 2021 Tuovinen S et al. *JAMA Network Open.*

**eTable 5.** Associations Between 25(OH)D and Developmental Milestones

| | N | Ages and Stages Questionnaire Total score | | Ages and Stages Questionnaire Total score ≤-1 SD | |
|---|---|---|---|---|---|
| | | **B (95% CI)/** | ***p*** | **OR (95% CI)** | ***p*** |
| **12-month follow-up** | | | | | |
| Model 1 | 613 | 0.32 (-0.28, 0.92) | 0.30 | 1.07 (0.85, 1.33) | 0.57 |
| Model 2 | 524 | 0.60 (-0.03, 1.24) | 0.06 | 1.00 (0.77, 1.27) | 0.95 |
| **24-month follow-up** | | | | | |
| Model 1 | 630 | 0.37 (-0.05, 0.79) | 0.09 | 1.07 (0.86, 1.34) | 0.54 |
| Model 2 | 538 | 0.33 (-0.11, 0.78) | 0.14 | 1.09 (0.84, 1.41) | 0.51 |

B and 95 % CI from Tobit regression analyses refer to change in ASQ Total raw scores per one SD unit change in 25(OH)D; OR and 95 % CI from logistic regression analyses show the odds of belonging to the group scoring ≤-1SD vs. >-1SD in ASQ per one SD unit change in 25(OH)D.

Model 1 is crude model.

Model 2. Adjusted for sex, length of gestation, duration of breastfeeding, age at follow-up, maternal age at delivery, maternal smoking and depressive symptoms at childbirth, and maternal educational level (missing values dummy-coded to their own category).

Abbreviation: B, unstandardized regression coefficient; OR, odds ratio; 95% CI, 95% confidence interval; SD, standard deviation; ASQ, Ages and Stages Questionnaire; 25(OH)D, 25-hydroxyvitamin D

© 2021 Tuovinen S et al. *JAMA Network Open.*

**eTable 6.** Associations Between 25(OH)D and Child Developmental Milestone Subscale Scores

| | Model 1 | | Model 2 | |
|---|---|---|---|---|
| **Subscales of developmental milestones** | **B (95% CI)** | ***p*** | **B (95% CI)** | ***p*** |
| **12-month follow-up** | | | | |
| Communication skills | 1.07 (0.94, 1.23) | 0.31 | 1.12 (0.96, 1.30) | 0.14 |
| Gross motor skills | 0.98 (0.86, 1.13) | 0.82 | 1.02 (0.87, 1.15) | 0.85 |
| Fine motor skills | 1.07 (0.93, 1.23) | 0.37 | 1.09 (0.93, 1.27) | 0.28 |
| Problem solving skills | 1.09 (0.95, 1.25) | 0.24 | 1.18 (1.01, 1.37) | 0.04 |
| Personal social skills | 1.09 (0.95, 1.25) | 0.21 | 1.13 (0.97, 1.32) | 0.12 |
| **24-month follow-up** | | | | |
| Communication skills | 1.17 (1.01, 1.35) | 0.03 | 1.14 (0.97, 1.34) | 0.11 |
| Gross motor skills | 1.09 (0.94, 1.27) | 0.25 | 1.13 (0.97, 1.32) | 0.13† |
| Fine motor skills | 1.13 (0.98, 1.30) | 0.09 | 1.16 (0.99, 1.35) | 0.07 |
| Problem solving skills | 0.99 (0.86, 1.13) | 0.86 | 0.97 (0.84, 1.12) | 0.67 |
| Personal social skills | 1.09 (0.95, 1.26) | 0.22 | 1.10 (0.94, 1.28) | 0.25 |

Bs and 95 % CIs from Ordinal logistic regression analyses refer to one step change in (ASQ) developmental milestones subdomain score per one SD unit change in 25(OH)D

Model 1 is crude model.

Model 2 is adjusted for sex, length of gestation, duration of breastfeeding, age at follow-up, maternal age at delivery, maternal smoking and depressive symptoms at childbirth, and maternal educational level (missing values dummy-coded to their own category).

© 2021 Tuovinen S et al. *JAMA Network Open.*

†Adjusted as in Model 2 excluding maternal depressive symptoms. Maternal depressive symptoms were not available for all children which led to a reduced number of participants in analyses and empty or extreme small cells (has no cases) in lower end at the gross motor skills domain.

Abbreviation: B, unstandardized regression coefficient; 95% CI, 95% confidence interval; ASQ, Ages and Stages Questionnaire; 25(OH)D, 25-hydroxyvitamin D

© 2021 Tuovinen S et al. *JAMA Network Open.*

**eTable 7.** Associations Between 25(OH)D and Social-Emotional Problems and Competencies

| | N | Social-emotional problems or competencies | | Social-emotional problems ≥1.5 SD/ competencies ≤-1.5 SD | |
|---|---|---|---|---|---|
| | | **B (95% CI)/** | ***p*** | **OR (95% CI)** | ***p*** |
| **Externalizing domain** | | | | | |
| Model 1 | 648 | 0.02 (-0.06, 0.10) | 0.82 | 1.18 (0.88, 1.57) | 0.26 |
| Model 2 | 556 | 0.05 (-0.03, 0.13) | 0.24 | 1.34 (0.97, 1.83) | 0.07 |
| **Internalizing domain** | | | | | |
| Model 1 | 649 | -0.02 (-0.10, 0.06) | 0.94 | 0.92 (0.69, 1.23) | 0.57 |
| Model 2 | 557 | -0.07 (-0.15, 0.02) | 0.14 | 0.88 (0.64, 1.19) | 0.40 |
| **Dysregulation domain** | | | | | |
| Model 1 | 652 | 0.00 (-0.08, 0.08) | 0.96 | 1.06 (0.74, 1.52) | 0.76 |
| Model 2 | 559 | -0.01 (-0.09, 0.08) | 0.89 | 1.02 (0.70, 1.50) | 0.90 |
| **Competencies** | | | | | |
| Model 1 | 649 | -0.05 (-0.13, 0.03) | 0.23 | 1.04 (0.78, 1.41) | 0.78 |
| Model 2 | 557 | -0.03 (-0.12, 0.05) | 0.43 | 0.89 (0.64, 1.24) | 0.48 |

B and 95 % CI from linear regression analyses refer to change in ITSEA domain scores in SD units per one SD unit change in 25(OH)D; OR and 95 % CI from logistic regression analyses show the odds of belonging to the group scoring ≥1.5 SD vs. <1.5 SD (problems domains)/ ≤-1.5 SD vs. >-1.5SD (competencies domain) in ITSEA domains per one SD unit change in 25(OH)D.

Model 1 is crude model.

© 2021 Tuovinen S et al. *JAMA Network Open.*

Model 2 is adjusted for sex, length of gestation, duration of breastfeeding, age at follow-up (missing values replaced with a mean of the sample), maternal age at delivery, maternal smoking and depressive symptoms at childbirth, and educational level (missing values dummy-coded to their own category).

Abbreviation: B, unstandardized regression coefficient; OR, odds ratio; 95% CI, 95% confidence interval; SD, standard deviation; ITSEA, Infant-Toddler Social and Emotional Assessment; 25(OH)D, 25-hydroxyvitamin D

© 2021 Tuovinen S et al. *JAMA Network Open.*

**eTable 8.** Associations Between 25(OH)D and Social-Emotional Problems and Competencies Subscale Scores

| | Model 1 | | Model 2 | |
|---|---|---|---|---|
| **Social-emotional problems and competencies subscale scores** | **B (95% CI)** | ***p*** | **B (95% CI)** | ***p*** |
| **Externalizing domain** | | | | |
| Activity/Impulsity | 0.01 (-0.07, 0.08) | 0.90 | 0.04 (-0.05, 0.13) | 0.42 |
| Aggression/defiance | 0.04 (-0.04, 0.12) | 0.32 | 0.05 (-0.04, 0.14) | 0.29 |
| Peer aggression | 0.01 (-0.07, 0.08) | 0.85 | 0.03 (-0.06, 0.12) | 0.50 |
| **Internalizing domain** | | | | |
| Depression/Withdrawal | -0.01 (-0.09, 0.07) | 0.78 | -0.00 (-0.09, 0.09) | 0.94 |
| General anxiety | -0.01 (-0.09, 0.06) | 0.72 | -0.05 (-0.14, 0.04) | 0.30 |
| Separation distress | -0.05 (-0.13, 0.03) | 0.20 | -0.09 (-0.18, 0.01) | 0.07 |
| Inhibition to novelty | 0.01 (-0.07, 0.09) | 0.83 | -0.05 (-0.14, 0.05) | 0.34 |
| **Dysregulation domain** | | | | |
| Negative emotionality | 0.01 (-0.07, 0.09) | 0.81 | -0.04 (-0.14, 0.05) | 0.34 |
| Sleep | -0.10 (-0.17, -0.02) | 0.02 | -0.09 (-0.18, 0.01) | 0.06 |
| Eating | 0.08 (0.00, 0.16) | 0.05 | 0.07 (-0.03, 0.16) | 0.15 |
| Sensory sensitivity | 0.06 (-0.01, 0.14) | 0.11 | 0.05 (-0.05, 0.14) | 0.34 |
| **Competence domain** | | | | |
| Compliance | -0.03 (-0.11, 0.05) | 0.49 | -0.02 (-0.11, 0.08) | 0.68 |
| Attention | -0.02 (-0.10, 0.06) | 0.65 | -0.05 (-0.15, 0.04) | 0.27 |

© 2021 Tuovinen S et al. *JAMA Network Open.*

| | | | | | |
|---|---|---|---|---|---|
| | Mastery motivation | -0.02 (-0.10, 0.06) | 0.66 | 0.02 (-0.07, 0.11) | 0.67 |
| | | **Model 1** | | **Model 2** | |
| **Social-emotional problems and competencies subscale scores** | | **B (95% CI)** | ***p*** | **B (95% CI)** | ***p*** |
| | Imitation/Play | -0.03 (-0.10, 0.05) | 0.52 | -0.00 (-0.09, 0.09) | 0.97 |
| | Empathy | -0.04 (-0.11, 0.04) | 0.38 | 0.05 (-0.04, 0.15) | 0.25 |
| | Prosocial peer relations | -0.05 (-0.12, 0.03) | 0.23 | -0.00 (-0.10, 0.09) | 0.96 |

B and 95 % CI from linear regression analyses refer to change in ITSEA subscale scores in SD units per one SD unit change in 25(OH)D.

Model 1 is crude model.

Model 2 is adjusted for sex, length of gestation, duration of breastfeeding, age at follow-up (missing values replaced with a mean of the sample), maternal age at delivery, maternal smoking and depressive symptoms at childbirth, and maternal educational level (missing values dummy-coded to their own category).

Abbreviation: B, unstandardized regression coefficient; 95% CI, 95% confidence interval; SD, standard deviation; ITSEA, Infant-Toddler Social and Emotional Assessment; 25(OH)D, 25-hydroxyvitamin D

© 2021 Tuovinen S et al. *JAMA Network Open.*
