# Supplementary material for: Effect of High-Dose vs Standard-Dose Vitamin D Supplementation on Neurodevelopment of Healthy Term Infants: A Randomized Clinical Trial
Source: JAMA Netw Open. 2021 Sep 8;4(9):e2124493. doi: 10.1001/jamanetworkopen.2021.24493 (PMC8427371; doi:10.1001/jamanetworkopen.2021.24493)
Supplement: Supplement 3. — Data Sharing Statement [file jamanetwopen-e2124493-s003.pdf]

Data Sharing Statement

Tuovinen. Effect of High-Dose vs Standard-Dose Vitamin D Supplementation on Neurodevelopment of Healthy Term Infants. *JAMA Netw Open*. Published September 08, 2021. doi:10.1001/jamanetworkopen.2021.24493

Data
Data available: No
